# Healthy dietary patterns, biological aging, and kidney stones: evidence from NHANES 2007–2018

**DOI:** 10.3389/fnut.2025.1538289

**Published:** 2025-03-25

**Authors:** Chengcheng Wei, Qian Yang, Jingke He, Yu Luo, Kun Han, Junkun Li, Shuai Su, Jindong Zhang, Hongxing Wang, Delin Wang

**Affiliations:** ^1^Department of Urology, The First Affiliated Hospital of Chongqing Medical University, Chongqing, China; ^2^Institute for Brain Science and Disease, Key Laboratory of Major Brain Disease and Aging Research (Ministry of Education), Chongqing Medical University, Chongqing, China; ^3^Chongqing Key Laboratory of Molecular Oncology and Epigenetics, The First Affiliated Hospital of Chongqing Medical University, Chongqing, China; ^4^Department of Urology, Xinqiao Hospital, Army Medical University, Chongqing, China; ^5^People's Hospital of Chongqing Liangjiang New Area, Chongqing, China

**Keywords:** healthy dietary pattern, biological aging, kidney stone, epidemiology, prospective cohort study

## Abstract

**Background:**

This study aims to investigate the comprehensive association between healthy dietary patterns, biological aging, and kidney stones in a large population-based cohort.

**Methods:**

We analyzed data from 6 cycles of NHANES surveys from 2007 to 2018 and included a total of 26,755 participants. The association was examined using logistic regression, restricted cubic splines, and a mediation model. Machine learning with Shapley Additive Explanations (SHAP) was used to determine the relative importance of dietary factors. Sensitivity analysis was conducted to validate the stability of the results.

**Results:**

A higher healthy dietary score was significantly associated with a reduced risk of kidney stone prevalence, as indicated by the Alternative Healthy Eating Index (AHEI) (OR = 0.76, 95% CI 0.69–0.84), the Dietary Approaches to Stop Hypertension Index (DASHI) (OR = 0.67, 95% CI 0.58–0.77), the Healthy Eating Index 2020 (HEI-2020) (OR = 0.80, 95% CI 0.72–0.89), and the Mediterranean Diet Index (MEDI) (OR = 0.81, 95% CI 0.73–0.89). Conversely, higher aging indicators were associated with an increased risk of kidney stones, including the following: Klemera-Doubal Method Age (KDMAge) (OR = 2.40, 95% CI 1.70–3.37) and Phenotypic Age (PhenoAge) (OR = 2.36, 95% CI 1.75–3.19). Mediation analyses suggested that aging indicators significantly mediated the relationship between healthy dietary patterns and kidney stones. Machine learning with SHAP revealed the relative importance of dietary patterns and specific dietary components in this association. The sensitivity analysis was largely consistent with the primary analyses.

**Conclusion:**

These findings provide valuable insights into the complex interplay between dietary patterns, biological aging, and the risk of kidney stone. Promoting healthy dietary patterns may be an effective strategy for kidney stone prevention, potentially through the modulation of biological aging processes.

## 1 Introduction

Kidney stones are common urological disorders with a rising global incidence, particularly in developed countries (1–3). It is estimated that approximately 1 in 10 individuals will experience this ailment during their lifetime (4, 5). Beyond causing severe pain, kidney stones impose a substantial financial burden on healthcare systems worldwide, accounting for billions of dollars in annual medical costs and lost productivity (6, 7).

The pathogenesis of kidney stones is driven by a complex interplay of environmental and genetic factors, with dietary components playing a pivotal role (8). High sodium and protein diets, combined with inadequate water intake, are recognized as significant contributors to kidney stone formation (9–11). Consequently, dietary modifications, such as increased hydration and adjusted intakes of calcium and oxalate, have been recognized as fundamental strategies for reducing recurrence rates (12–14). Moreover, nutritional research favors dietary scores over discrete dietary components, as they provide a comprehensive understanding of dietary patterns and their health impacts (15). Globally, various dietary scores—including the Alternative Healthy Eating Index (AHEI), the DASH Index in serving sizes from the DASH trial (DASHI), the Healthy Eating Index 2020 (HEI-2020), and the and MED Index in serving sizes from the PREDIMED trial (MEDI)—are used to assess the complex interactions between dietary nutrition, disease risks, and metabolic disorders, with an emphasis on dietary recommendations (16). These findings highlight the necessity of rigorous research into the diet–kidney stone relationships to develop robust preventive and therapeutic interventions.

Moreover, the pathogenesis of kidney stones is closely related to chronic conditions such as obesity (17), diabetes (18), and cardiovascular disease (19), necessitating multifaceted health interventions for prevention and management. Age notably influences the progression of these conditions, as declining metabolic rates with advancing age change the body’s ability to process dietary minerals, potentially increasing the risk of kidney stone. Research confirms that biological age provides a more accurate representation of an individual’s physiological state than chronological age. Algorithms such as KDMAge and PhenoAge, which assess biological age based on clinical markers, have proven highly accurate in predicting morbidity and mortality rates (20–25). KDMAge and PhenoAge were chosen for their comprehensive assessment of biological aging and predictive power for chronic diseases. Their associated biomarkers, such as creatinine and albumin, are linked to kidney function and metabolic disturbances, suggesting potential relationships with kidney stone formation through mechanisms such as inflammation and oxidative stress. Despite data indicating a higher occurrence of kidney stones among individuals aged 20–40 years (26), large-scale studies examining the influence of biological age on kidney stones remain limited. Therefore, exploring the specific roles of dietary patterns and biological age in the risk of kidney stone is important for enhancing preventive and therapeutic approaches.

In this context, our study used the US National Health and Nutrition Examination Survey (NHANES) database to systematically investigate the association between healthy dietary patterns, biological aging, and the prevalence of kidney stones. The development of kidney stones is influenced by a variety of environmental factors, with dietary components playing a crucial role. However, the association between dietary factors and kidney stones remains unclear. This study aims to investigate the relationship between healthy dietary patterns, biological aging, and the prevalence of kidney stones in a large population-based cohort.

## 2 Methods

### 2.1 Study population and design

**Figure 1:**
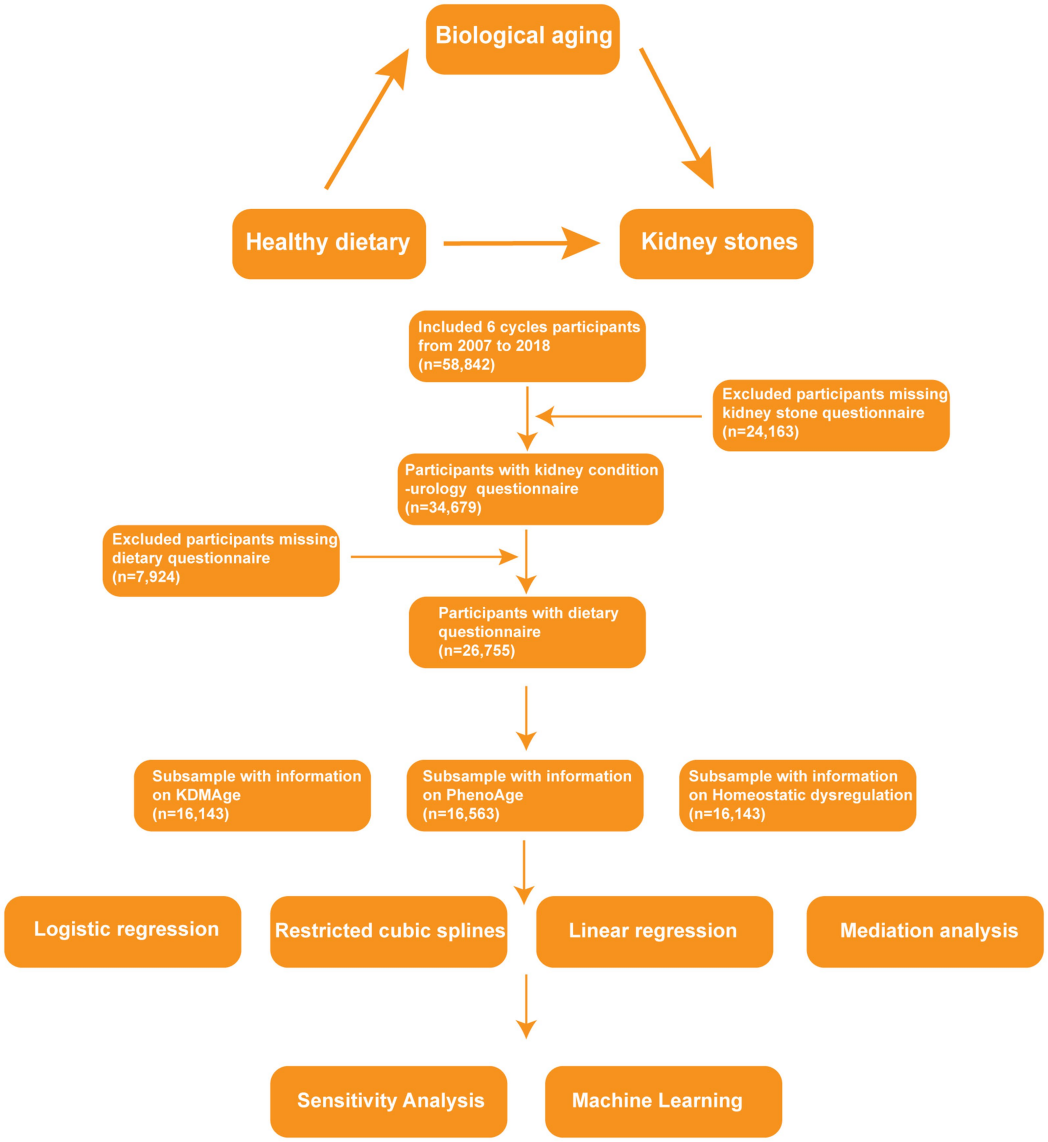
Flowchart of this study.

The National Center for Health Statistics (NCHS) conducts the NHANES research project, a nationwide study assessing the health and nutritional status of both adults and children in the United States. The study is overseen by the Centers for Disease Control and Prevention (CDC). The NHANES survey data are publicly accessible on the official webpage: https://www.cdc.gov/nchs/nhanes/about_nhanes.htm. Our analysis includes cross-sectional data gathered from six NHANES study cycles (2007–2018), which initially comprised 58,842 participants. A total of 24,163 individuals were excluded due to incomplete kidney stone questionnaire data. Subsequently, we excluded 7,924 individuals with dietary survey data. After these exclusions, a final sample of 26,755 individuals remained for additional research (Figure 1). Finally, a subsample was analyzed based on indicator data that were available again, comprising 16,143 individuals for KDMAge and homeostatic dysregulation and 16,563 individuals for PhenoAge. Since NHANES data were used for our investigation, and the NHANES procedures were previously approved by the NCHS Research Ethics Review Board (ERB) and the NHANES Institutional Review Board (IRB), no further approvals were required. The study adhered to the ethical principles outlined in the Declaration of Helsinki and was reported in accordance with the Strengthening The Reporting Of Cohort Studies in Surgery (STROCSS) criteria.

### 2.2 Aging indicators assessment

We measured biological aging utilizing the “BioAge” package with information from NHANES (27). This package computes three biological aging metrics: Klemera-Doubal Method (KDM) biological age (KDMAge), phenotypic age (PhenoAge), and homeostatic dysregulation, based on established biomarker algorithms. The KDM biological age forecast for an individual indicates the age at which their physiology is expected to align with typical norms. The KDM was assessed using the “Levine Original” KDM algorithm, which relies on chronological age and 10 biomarkers, namely total cholesterol, systolic blood pressure, albumin, alkaline phosphatase, blood urea nitrogen, creatinine, C-reactive protein, cytomegalovirus optical density, hemoglobin A1C (HbA1c), and forced expiratory volume in 1 s (FEV1). Nine biomarkers comprise the “Levine Original” PhenoAge, alongside chronological age: albumin, alkaline phosphatase, creatinine, C-reactive protein, fasting glucose, white blood cell count, lymphocyte percentage, mean cell volume, and red cell distribution width (28). The homeostatic dysregulation value for an individual indicates the extent to which their physiology deviates from that of a healthy reference. Higher levels of homeostatic dysregulation suggest an accelerated stage of biological aging and an increased risk of illness, disability, and mortality. Conversely, lower levels of homeostatic dysregulation signify a delayed biological aging process and a reduced likelihood of illness, incapacity, and death. Homeostatic dysregulation is calculated using the Mahalanobis distance, which evaluates the deviation of a set of biomarkers from a reference sample. The Mahalanobis distance formula is structured as follows (29):


$$D_{M}\left(\vec{x}\right)=\sqrt{{\left(\vec{x}-\vec{\mu }\right)}^{T}S^{-1}\left(\vec{x}-\vec{\mu }\right)}$$

This is equivalent to normalizing each biomarker by its variance and then summing the squared deviance for each observation, assuming that all variables are uncorrelated:


$$D_{M}\left(\vec{x}\right)=\sqrt{\overset{n}{\underset{i=1}{\sum }}\frac{{\left(x_{i}-\mu_{i}\right)}^{2}}{\sigma^{2}\left(x_{i}\right)}}q$$

### 2.3 Kidney stone assessment

Kidney stone status was extracted from the “Kidney Conditions—Urology” section of the NHANES Questionnaire Data. Trained interviewers used the Computer-Assisted Personal Interview (CAPI) system to ask interview subjects at home whether they had ever experienced kidney stones (KIQ026). Individuals who responded “yes” were classified as having a history of kidney stones.

### 2.4 Assessment of dietary scores

A validated semiquantitative food frequency questionnaire (FFQ) consisting of over 130 questions was used to collect dietary data, distributed every 2–4 years. Detailed descriptions of the FFQs’ validity and repeatability are available, demonstrating a strong correlation between the nutrients measured by the FFQs and multiple weeks of food records or dietary biomarkers. Using the food and nutrient components, we calculated the dietary scores for the AEHI, DASHI, HEI-2020, and MEDI to reflect adherence to healthy eating. For these four scores, a better diet is indicated by a higher score, and a less healthy diet is indicated by a lower score. In 2002, the Alternate Healthy Eating Index (AHEI) was introduced as an alternative to the HEI, which was based on dietary components and nutrients that were indicative of the risk of chronic diseases (30). Emphasizing increased consumption of fruits, vegetables, and low-fat dairy products while reducing total and saturated fats, the DASH diet significantly lowers blood pressure and is recommended for the prevention of cardiovascular diseases (31). The HEI-2020 is a revised and updated version of the HEI based on the 2020–2025 Dietary Guidelines for Americans (DGA), which includes 13 components aiming to maintain a healthy body weight and minimize the risk of chronic diseases (32). The Mediterranean diet is recognized for its ability to identify overall dietary patterns rather than just single foods or nutrients, which is considered effective in providing a considerable defense against mortality, the development of heart diseases, and major chronic degenerative diseases (33).

### 2.5 Covariates

Data from the laboratory, questionnaires, and demographics were used to collect covariates. Using a standardized questionnaire, we included variables such as age, sex, race, marital status, education level, poverty-income ratio, obesity, smoking, alcohol use, diabetes, high blood pressure, food insecurity, water consumption, serum calcium, and sitting time. Age, sex, and obesity were identified as adjustment factors due to their roles as confounders in the relationship between dietary habits and kidney stone formation. Prior studies and literature reviews confirm the significance of these variables in influencing the risk of kidney stone. A body mass index of 30 kg/m^2^ was considered obesity. Participants were defined as smokers if they had smoked at least 100 cigarettes in their lifetime. Participants were defined as alcohol users if they had consumed at least 12 alcoholic drinks in 1 year. In evaluating food security, we utilized data from the “Food Security” questionnaire. The assessment was conducted using the 10-item U.S. Food Security Survey Module available at https://aspe.hhs.gov/topics/poverty-economic-mobility/poverty-guidelines/prior-hhs-poverty-guidelines-federal-register-references. An acknowledgment of six or more affirmative responses signifies significant food insecurity, whereas fewer than six responses indicate marginal food security or food security. Diagnosed diabetes was defined as a fasting glucose level of 7 mmol/L (126 mg/dL), an HbA1c level of 6.5%, or a diabetes diagnosis. A systolic blood pressure of 130 mm Hg, a diastolic blood pressure of 80 mm Hg, or the use of antihypertensive drugs was considered high blood pressure. Water consumption data were obtained from the dietary questionnaire. Serum calcium was obtained from the laboratory data of the standard biochemistry profile. Sitting time was derived from the questionnaire data on physical activity. Each of these variables is described on the NHANES website.

### 2.6 Statistical analysis

Sample weights, clustering, and stratification were all considered while evaluating the data due to the complex sampling method of the NHANES. We evaluated the relationships between kidney stones, aging indicators, and dietary scores using multivariable logistic regression models. Non-linear relationships were analyzed with restricted cubic splines. The relationship between healthy dietary scores and aging indicators was investigated using multiple linear regression models. We utilized the R package “mediation” to assess the direct effect (DE), indirect effect (IE), and total effect (TE) for causal mediation research (34). The “shapviz” package was used to build the machine learning model to predict the relative importance of dietary patterns and components. R software (version 4.0.1) was used for all statistical analyses, with a significance threshold set at *p* < 0.05 (two-sided).

## 3 Results

### 3.1 Population characteristics

Table 1 provides an overview of the baseline characteristics of participants classified by kidney stone status, while Supplementary Table S1 presents the survey-weighted descriptive statistics for these participants. We included 26,755 participants from the NHANES survey conducted between 2007 and 2018, all of whom met the research criteria for our study. The median age of participants was 50.14 ± 17.61 years. Among these participants, 2,577 were kidney stone patients, while 24,178 were participants without kidney stones. We observed varying dietary scores, including the Alternative Healthy Eating Index (AHEI), the DASH Index from the DASH trial (DASHI), the HEI-2020, and the MED Index from the PREDIMED trial (MEDI), all of which displayed different distribution patterns between kidney stone and non-kidney stone participants. Significantly lower dietary scores were noted among kidney stone participants. Moreover, kidney stone participants exhibited higher levels of aging indicators, including KDMAge, PhenoAge, KDMAge accelerated aging, PhenoAge accelerated aging, and indicators of homeostatic dysregulation. We also found notable differences across several variables between kidney stone participants and non-participants, including age, sex, race, marital status, obesity, smoking, alcohol use, diabetes, high blood pressure, food insecurity, and water consumption (*p* < 0.05).

**Table 1:** Basic characteristics of participants by kidney stone among U.S. adults in NHANES 2007–2018.

Characteristics	All (*n* = 26,755)	Kidney stone (*n* = 2,577)	Non-kidney stone (*n* = 24,178)	*p*-value
Age (mean ± SD)	50.14 ± 17.61	56.42 ± 16.00	49.47 ± 17.64	<0.001
Sex (*N*, %)				<0.001
Male	12,742 (47.62%)	1,426 (55.34%)	11,316 (46.80%)	
Female	14,013 (52.38%)	1,151 (44.66%)	12,862 (53.20%)	
Race (*N*, %)				<0.001
Hispanic	6,582 (24.60%)	597 (23.17%)	5,985 (24.75%)	
Non-Hispanic White	11,456 (42.82%)	1,447 (56.15%)	10,009 (41.40%)	
Non-Hispanic Black	5,803 (21.69%)	346 (13.43%)	5,457 (22.57%)	
Other races	2,914 (10.89%)	187 (7.26%)	2,727 (11.28%)	
Education (*N*, %)				0.45
Less than high school education	6,098 (22.81%)	603 (23.41%)	5,495 (22.75%)	
High school graduate or higher	20,631 (77.19%)	1973 (76.59%)	18,658 (77.25%)	
Marital status (*N*, %)				<0.001
Not married	12,871 (48.13%)	1,081 (41.98%)	11,790 (48.78%)	
Married or living with a partner	13,872 (51.87%)	1,494 (58.02%)	12,378 (51.22%)	
Poverty income ratio (mean ± SD)	2.53 ± 1.63	2.52 ± 1.60	2.53 ± 1.63	0.867
Obesity (*N*, %) &	10,485 (39.59%)	1,188 (46.64%)	9,297 (38.84%)	<0.001
Smoking (*N*, %) ^	11,798 (44.10%)	1,310 (50.83%)	10,488 (43.38%)	<0.001
Alcohol use (*N*, %) ^^	17,047 (68.26%)	1,616 (66.01%)	15,431 (68.50%)	0.012
Diabetes (*N*, %) *	4,690 (20.08%)	717 (31.64%)	3,973 (18.83%)	<0.001
High blood pressure (*N*, %) **	14,035 (53.60%)	1,665 (65.60%)	12,370 (52.31%)	<0.001
Food insecurity (*N*, %)	2,323 (8.79%)	264 (10.37%)	2059 (8.63%)	0.003
Water consumption (g) (mean ± SD)	1034.88 ± 962.93	993.31 ± 980.92	1039.31 ± 960.91	<0.001
Serum calcium (mg/dL) (mean ± SD)	9.39 ± 0.37	9.38 ± 0.39	9.39 ± 0.37	0.141
Sitting time (*N*, %)				0.625
<240 min/day	10,842 (40.71%)	1,020 (39.80%)	9,822 (40.81%)	
240–360 min/day	5,822 (21.86%)	556 (21.69%)	5,266 (21.88%)	
360–480 min/day	4,668 (17.53%)	455 (17.75%)	4,213 (17.50%)	
≥480 min/day	5,299 (19.90%)	532 (20.76%)	4,767 (19.81%)	
Dietary score (mean ± SD)				
AEHI	38.57 ± 11.49	37.94 ± 11.07	38.64 ± 11.53	0.003
DASHI	3.56 ± 1.18	3.43 ± 1.15	3.57 ± 1.19	<0.001
HEI-2020	51.66 ± 12.03	50.45 ± 11.49	51.79 ± 12.08	<0.001
MEDI	3.58 ± 1.04	3.47 ± 1.00	3.59 ± 1.05	<0.001
Indicators of aging $				
KDMAge, years (mean ± SD)	48.30 ± 17.19	54.82 ± 16.06	47.58 ± 17.16	<0.001
PhenoAge, years (mean ± SD)	48.53 ± 19.93	56.48 ± 18.71	47.66 ± 19.86	<0.001
KDMAge accelerated aging (mean ± SD)	−2.20 ± 6.52	−1.89 ± 6.90	−2.23 ± 6.47	0.048
PhenoAge accelerated aging (mean ± SD)	−1.94 ± 6.93	−0.26 ± 7.58	−2.12 ± 6.83	<0.001
KDMAge accelerated aging (*N*, %)	5,482 (33.96%)	586 (36.62%)	4,896 (33.67%)	0.018
PhenoAge accelerated aging (*N*, %)	5,147 (31.08%)	676 (41.42%)	4,471 (29.94%)	<0.001
Homeostatic dysregulation (mean ± SD)	2.22 ± 0.98	2.44 ± 1.05	2.20 ± 0.97	<0.001

The data are shown as *N* (%) or the mean. The Kruskal-Wallis rank sum test was employed to establish if it is a continuous variable. Using Fisher’s exact probability test, the *p*-value for continuous variables with a theoretical value of less than 10 was found. Weighted chi-square was used to get the *p*-value for categorical data. The values are shown as means ± SD or percentage. SD, standards deviation.

& Body mass index of 30 kg/m^2^ was considered obesity.

^ Participants were classified as smokers if they had smoked at least 100 cigarettes in their lifetime.

^^ Participants were considered alcohol users if they had at least 12 alcoholic drinks in 1 year.

* Diagnosed diabetes was defined as a fasting glucose level of 7 mmol/L (126 mg/dL), a hemoglobin A1c level of 6.5%, or a diagnosed diabetes.

** Systolic blood pressure of 130 mm Hg, diastolic blood pressure of 80 mm Hg, or the use of antihypertensive drugs was considered high blood pressure.

$ Accelerated biological aging was defined as a KDMAge accelerated aging or PhenoAge accelerated aging greater than 0, whereas non-accelerated aging was defined as a KDMAge accelerated aging or PhenoAge accelerated aging less than or equal to 0.

### 3.2 Association of healthy dietary scores and kidney stone prevalence risk

**Figure 2:**
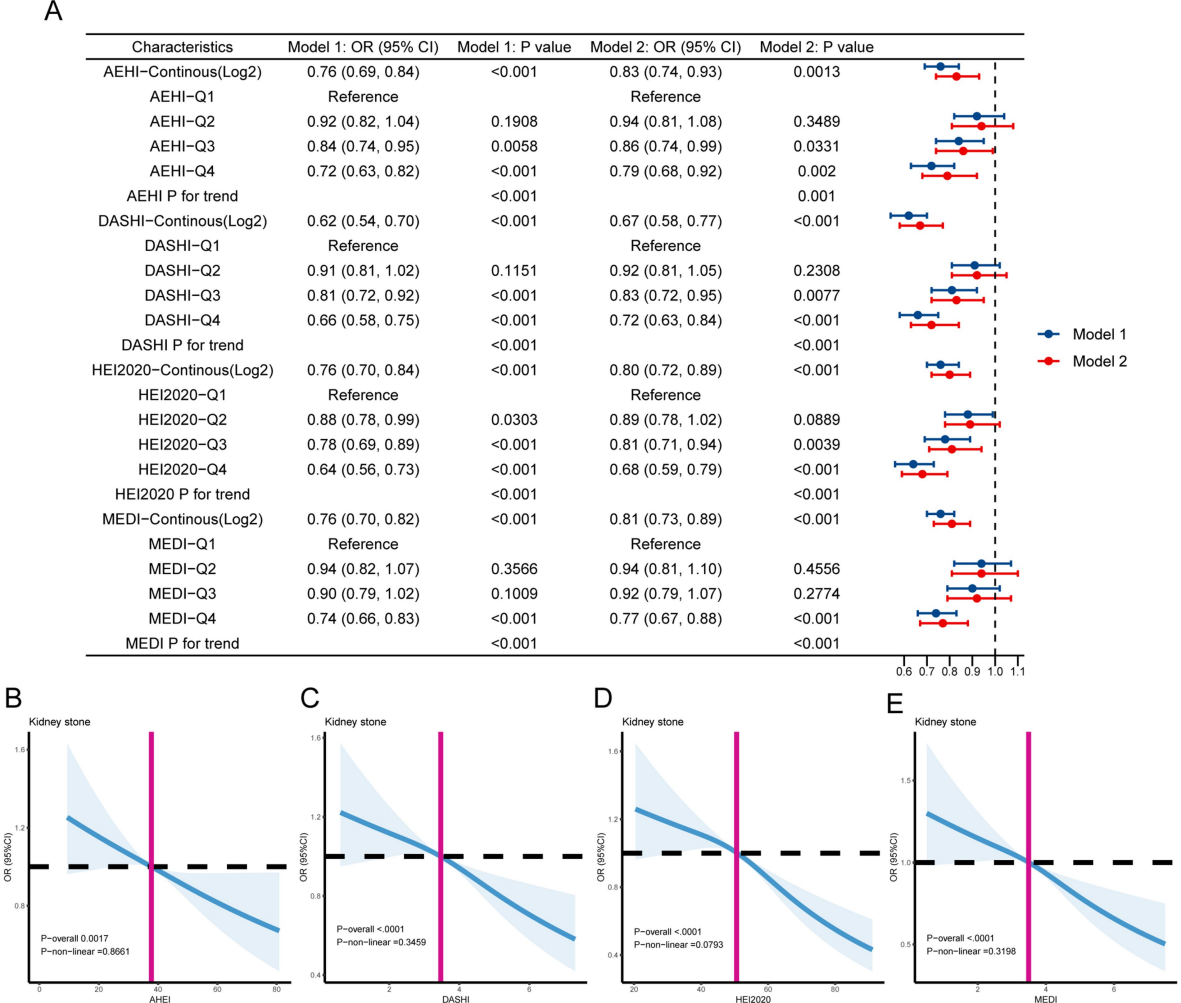
**(A)** The association between healthy dietary scores and kidney stones is depicted using forest plots. Model 1 was adjusted for age, sex, race, marital status, education level, and poverty income ratio. Model 2 was adjusted for age, sex, race, marital status, education level, poverty income ratio, obesity, smoking, alcohol use, diabetes, high blood pressure, food insecurity, water consumption, serum calcium, and sitting time. **(B–E)** The linear associations between kidney stones and healthy dietary scores are depicted using restricted cubic splines. The solid lines represent the odds ratios (ORs) based on the restricted cubic splines of healthy dietary scores. The top and lower 95% confidence interval (CI) boundaries are shaded. The adjustment factors are the same as those used in the expanded model. The model is adjusted for age, sex, race, marital status, educational level, poverty income ratio, obesity, smoking, alcohol use, diabetes, high blood pressure, food insecurity, water consumption, serum calcium, and sitting time.

We used the multiple logistic regression analysis to explore the potential relationship between healthy dietary scores and kidney stones (Figure 2A). The log2-transformed exposures, including the AHEI (OR = 0.76, 95% CI 0.69–0.84, *p* < 0.001), DASHI (OR = 0.67, 95% CI 0.58–0.77, *p* < 0.001), HEI-2020 (OR = 0.90, 95% CI 0.72–0.89, *p* < 0.001), and MEDI (OR = 0.81, 95% CI 0.73–0.89, *p* < 0.001), showed a statistically significant negative correlation with kidney stones in the fully adjusted models. The fourth quartile of dietary scores indicated significantly lower odds of kidney stones compared to the first quartile. In all models, a monotonically increasing trend in kidney stone prevalence risk was observed as the quartiles of healthy dietary scores increased (all *p* for trend <0.01) (Figure 2A). Additionally, to explore the potential non-linear association between healthy dietary scores and the occurrence of kidney stones, we conducted a restricted cubic spline model. The results indicate linear relationships between healthy dietary scores and kidney stones (*P*-overall <0.05 and *p* for non-linearity >0.05) (Figures 2B–E). We also analyzed the characteristics of specific dietary components (Supplementary Table S9) and the association of these components with kidney stones (Supplementary Table S10).

### 3.3 Association between aging indicators and kidney stone

In Table 2, a multiple logistic regression analysis was used to demonstrate the associations between biological aging and kidney stone incidence. We found significant positive associations between kidney stones and aging indicators, including KDMAge (OR = 2.40, 95% CI 1.70–3.37, *p* < 0.0001) and PhenoAge (OR = 2.36, 95% CI 1.75–3.19, *p* < 0.0001) in Model 2. However, no statistical difference was detected regarding homeostatic dysregulation in the fully adjusted model (OR = 1.11, 95% CI 0.97–1.27, *p* = 0.1421). The risk of kidney stones increased monotonically with higher quartiles of KDMAge and PhenoAge. The results indicated that accelerated aging was associated with a greater risk of kidney stones: KDMAge (OR = 1.20, 95% CI 1.04–1.38, *p* = 0.0104) and PhenoAge (OR = 1.89, 95% CI 1.16–3.06, *p* = 0.0104). Additionally, the linear association between kidney stones and aging markers was examined using cubic spline curves (Supplementary Figure S1), which demonstrated non-linear correlations. However, no association was found between kidney stones and homeostatic dysregulation (*p* overall >0.05).

**Table 2:** Multiple logistic regression analysis revealed the associations of aging indicators with kidney stones in adults.

Exposure		Model 1		Model 2	
		Odds ratio (95% CI)	*p*-value	Odds ratio (95% CI)	*p*-value
KDMAge	Continuous (Log2)	3.25 (2.47, 4.28)	<0.0001	2.40 (1.70, 3.37)	<0.0001
	Q1				
	Q2	1.97 (1.57, 2.47)	<0.0001	1.61 (1.22, 2.12)	0.0007
	Q3	2.56 (1.89, 3.46)	<0.0001	1.91 (1.32, 2.77)	0.0006
	Q4	2.82 (1.91, 4.15)	<0.0001	1.84 (1.15, 2.93)	0.0111
	Non-accelerated aging	Reference		Reference	
	Accelerated aging	1.34 (1.19, 1.51)	<0.0001	1.20 (1.04, 1.38)	0.0104
PhenoAge	Continuous (Log2)	3.22 (2.53, 4.12)	<0.0001	2.36 (1.75, 3.19)	<0.0001
	Q1	Reference		Reference	
	Q2	2.02 (1.61, 2.53)	<0.0001	1.76 (1.33, 2.32)	<0.0001
	Q3	2.69 (1.98, 3.65)	<0.0001	1.98 (1.36, 2.88)	0.0004
	Q4	3.12 (2.09, 4.65)	<0.0001	1.89 (1.16, 3.06)	0.0103
	Non-accelerated aging	Reference		Reference	
	Accelerated aging	1.51 (1.34, 1.69)	<0.0001	1.24 (1.07, 1.44)	0.0042
Homeostatic dysregulation	Continuous (Log2)	1.33 (1.19, 1.49)	<0.0001	1.11 (0.97, 1.27)	0.1421
	Q1	Reference		Reference	
	Q2	1.18 (0.99, 1.40)	0.0607	1.08 (0.88, 1.33)	0.4651
	Q3	1.35 (1.13, 1.61)	0.0008	1.24 (1.00, 1.54)	0.0488
	Q4	1.55 (1.29, 1.87)	<0.0001	1.23 (0.97, 1.55)	0.0875

Model 1 was adjusted for age, sex, race, marital status, education level, and poverty income ratio. Model 2 was adjusted for age, sex, race, marital status, education level, poverty income ratio, obesity, smoking, alcohol use, diabetes, high blood pressure, food insecurity, water consumption, serum calcium, and sitting time.

Non-accelerated aging means that the residual of the regression of KDMAge or PhenoAge based on chronological age is ≤ 0.

Accelerated aging means that the residual of the regression of KDMAge or PhenoAge based on chronological age is > 0.

### 3.4 Association between healthy dietary scores and aging indicators

**Figure 3:**
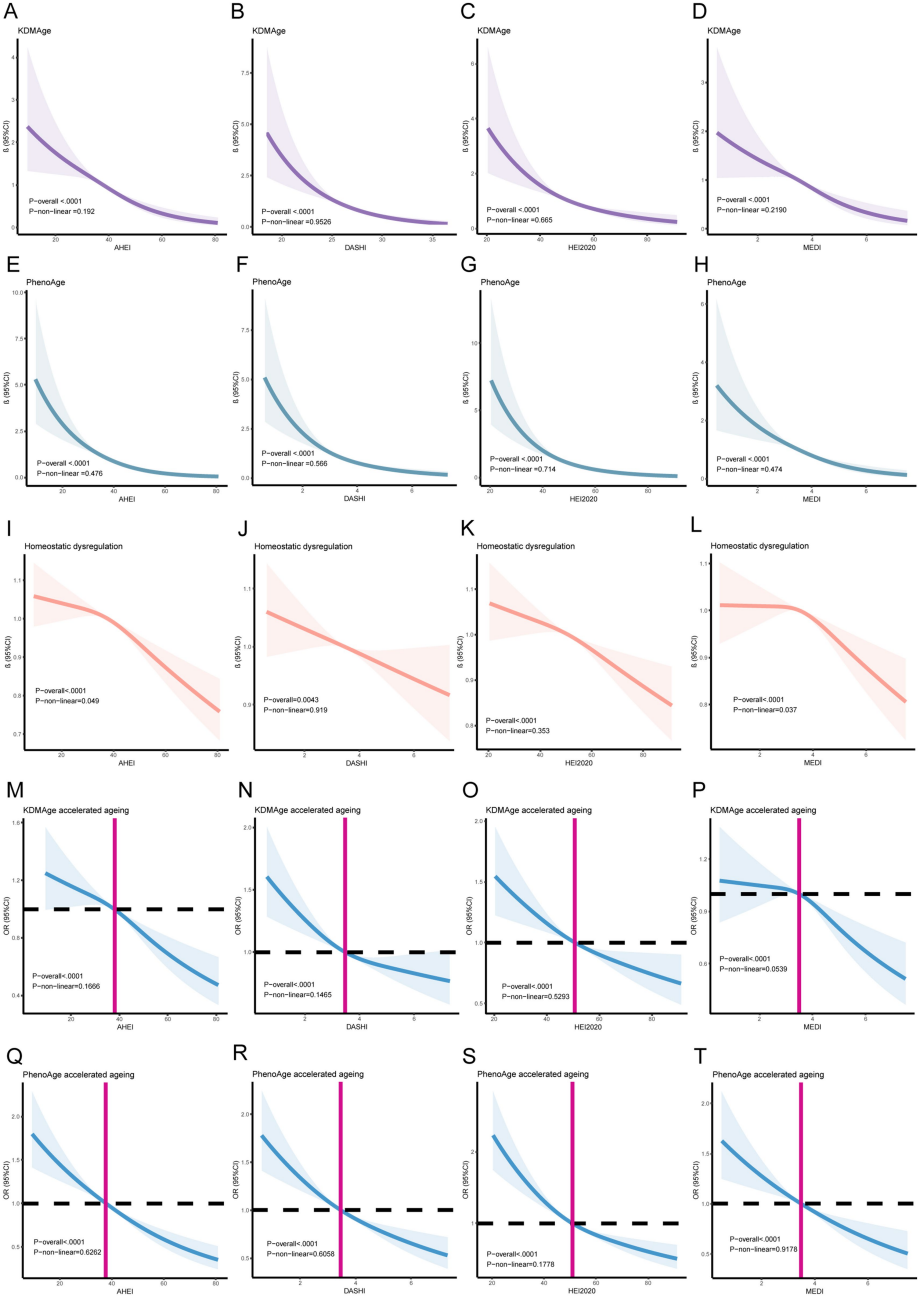
Linear and Non-linear relationships between healthy dietary scores and aging indicators/accelerating aging using restricted cubic splines. **(A)** The AHEI and KDMAge; **(B)** DASHI and KDMAge; **(C)** the HEI-2020 and KDMAge; **(D)** the MEDI and KDMAge; **(E)** the AHEI and PhenoAge; **(F)** DASHI and PhenoAge; **(G)** the HEI-2020 and PhenoAge; **(H)** the MEDI and PhenoAge; **(I)** the AHEI and Homeostatic dysregulation; **(J)** the DASHI and Homeostatic dysregulation; **(K)** the HEI-2020 and Homeostatic dysregulation; **(L)** the MEDI and Homeostatic dysregulation; **(M)** the HEI-2020 and KDMAge accelerated aging; **(N)** the DASHI and KDMAge accelerated aging; **(O)** the HEI-2020 and KDMAge accelerated aging; **(P)** the MEDI and KDMAge accelerated aging; **(Q)** the AHEI and PhenoAge accelerated aging; **(R)** DASHI and PhenoAge accelerated aging; **(S)** the HEI-2020 and PhenoAge accelerated aging; **(T)** the MEDI and PhenoAge accelerated aging. Generalized-linear models and logistic regression models for age, sex, race, marital status, education level, poverty income ratio, obesity, smoking, alcohol use, diabetes, high blood pressure, food insecurity, water consumption, serum calcium, and sitting time.

We conducted an analysis to examine the association between healthy dietary scores and aging indicators listed in Supplementary Table S2. After adjusting for all covariates, we observed significant negative associations between healthy dietary scores (the AHEI, DASHI, HEI-2020, and MEDI) and aging indicators (KDMAge, PhenoAge, homeostatic dysregulation, KDMAge accelerated aging, and PhenoAge accelerated aging). Additionally, the results of the multiple logistic regression analysis indicated that higher healthy dietary scores were associated with a decreased risk of accelerated aging, as shown by KDMAge and PhenoAge. Restricted cubic splines in Figure 3 visualize both linear and non-linear relationships between healthy dietary scores and aging indicators. KDMAge, PhenoAge, KDMAge accelerated aging, and PhenoAge accelerated aging all exhibited linear associations with the four dietary scores.

### 3.5 Aging indicators partially mediated the association between a healthy diet and kidney stones

**Figure 4:**
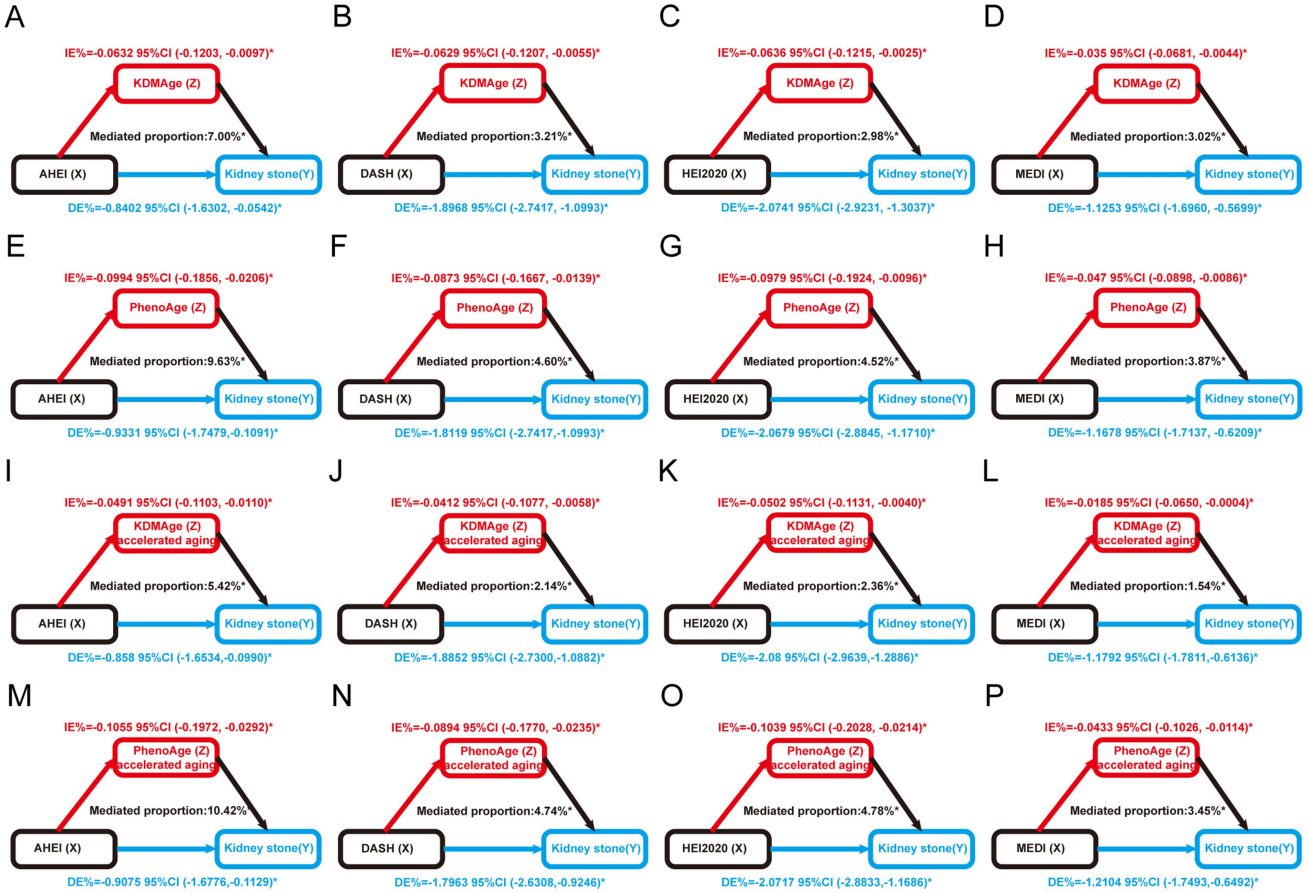
Estimated proportion of the association between healthy dietary scores and kidney stones mediated by aging markers. **(A)** The AHEI mediated by KDMAge; **(B)** the DASHI mediated by KDMAge; **(C)** the HEI-2020 mediated by KDMAge; **(D)** the MEDI mediated by KDMAge; **(E)** the AHEI mediated by PhenoAge; **(F)** the DASHI mediated by PhenoAge; **(G)** the HEI-2020 mediated by PhenoAge; **(H)** the MEDI mediated by PhenoAge; **(I)** the AHEI mediated by KDMAge accelerated aging; **(J)** the DASHI mediated by KDMAge accelerated aging; **(K)** the HEI-2020 mediated by KDMAge accelerated aging; **(L)** the MEDI mediated by KDMAge accelerated aging; **(M)** the AHEI mediated by PhenoAge accelerated aging; **(N)** the DASHI mediated by PhenoAge accelerated aging; **(O)** the HEI-2020 mediated by PhenoAge accelerated aging; **(P)** the MEDI mediated by PhenoAge accelerated aging. Age, sex, race, marital status, education level, poverty income ratio, obesity, smoking, alcohol use, diabetes, high blood pressure, food insecurity, water consumption, serum calcium, and sitting time were all considered while adjusting the models. IE, indirect effect; DE, direct effect; Proportion of mediation = IE / (DE + IE); *p < 0.05.

We assessed the mediating effect in the association between kidney stones and a healthy diet using mediation analysis, with aging indicators as mediators (Supplementary Table S3). Our results suggest that the aging indicators KDMAge, PhenoAge, KDMAge accelerated aging, and PhenoAge accelerated aging significantly mediated the associations between healthy dietary patterns (the AHEI, DASHI, HEI-2020, and MEDI) and the risk of kidney stones (Figure 4). For KDMAge and healthy dietary patterns (the AHEI, DASHI, HEI-2020, and MEDI), the mediation proportions were 7.00, 3.21, 2.98, and 3.02%, respectively. For PhenoAge and healthy dietary patterns (the AHEI, DASHI, HEI-2020, and MEDI), the mediation proportions were 9.63, 4.60, 4.52, and 3.87%, respectively. For PhenoAge accelerated aging and healthy dietary patterns (the AHEI, DASHI, HEI-2020, and MEDI), the mediation proportions were 10.42, 4.74, 4.78, and 3.45%, respectively. Nevertheless, no significant mediation effects were observed in the associations between healthy dietary scores and kidney stones mediated by homeostatic dysregulation (all *p* > 0.05).

### 3.6 Machine learning identify the relative importance

**Figure 5:**
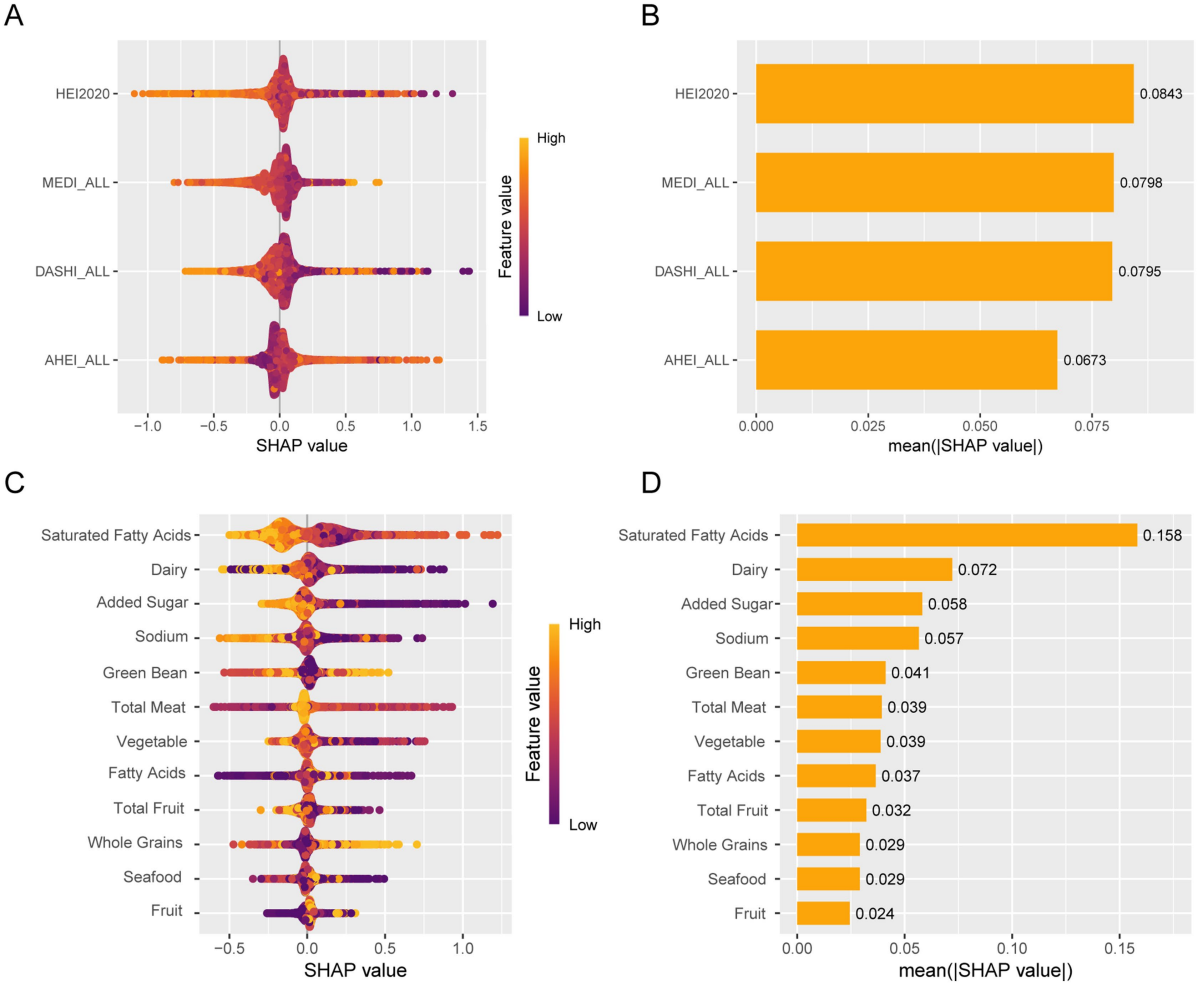
Machine learning using SHAP identifies relative importance. **(A)** Bee warm SHAP analysis of dietary patterns. **(B)** Importance of the SHAP representation of dietary patterns. **(C)** Bee warm SHAP analysis of specific dietary components. **(D)** Bee warm SHAP analysis of specific dietary components.

Furthermore, we constructed the SHAP model for machine learning to identify the relative importance of dietary patterns and specific dietary components (HEI-2020). We found that HEI-2020 was the most important dietary score for kidney stones compared to other dietary scores. Regarding specific dietary components, saturated fatty acids were the most important component for kidney stones (Figure 5).

### 3.7 Sensitivity analysis

Initially, we conducted multiple imputations to complete the cases. We analyzed the association between healthy dietary scores and the risk of prevalent kidney stones using complete cases (Supplementary Table S4). Additionally, we carried out multiple logistic regression to identify the associations between indicators of aging and kidney stones in adults (Supplementary Table S5) and further examined the associations between indicators of aging and kidney stones within the complete cases (Supplementary Table S6). The relationships observed in the complete case analysis were largely consistent with those from the primary analyses. Subsequently, we conducted a sensitivity analysis stratified by two 6-year survey periods (2007–2012 and 2013–2018) to evaluate the consistency of associations between healthy dietary scores, indicators of aging, and kidney stones (Supplementary Tables S7, S8).

## 4 Discussion

In this study, we used the NHANES database to elucidate the relationship between healthy dietary practices and the prevalence of kidney stones. Our analysis revealed a robust linear negative association between healthy diet scores and the risk of kidney stone prevalence. Conversely, biological aging and accelerated aging showed a significant association with a higher prevalence of kidney stones, exhibiting linear correlations. Notably, the aging indicators KDMAge and PhenoAge showed significant statistical differences when mediated by the four dietary scores (the AHEI, HEI-2020, MEDI, and DASHI), highlighting the mediating role of KDMAge and PhenoAge in the relationship between healthy diets and kidney stones. A similar association was also observed in cases of accelerated aging. This finding highlights the potential for a healthy diet to reduce the risk of kidney stone and slow disease progression by decelerating biological aging. Machine learning SHAP identified saturated fatty acids as the most significant component associated with kidney stones.

Our findings align with prior research, which consistently demonstrates that healthier dietary patterns, as represented by dietary indices such as the AEHI, DASHI, HEI-2020, and MEDI, are associated with a reduced risk of kidney stones, emphasizing the critical role of dietary habits in prevention and management (35, 36). These dietary indices prioritize a balanced intake of vegetables, nuts, whole grains, and low-fat dairy products, which are crucial for preventing both chronic metabolic disorders and kidney stone formation (30, 31, 32, 33). Diets rich in fruits and vegetables provide key nutrients such as citrate, potassium, and magnesium, which lower urinary calcium levels and inhibit the formation of oxalate crystals, thereby reducing the risk of kidney stone (37, 38, 39, 40). In contrast, high-protein diets, particularly those with excessive red meat and seafood, elevate the urinary levels of calcium, uric acid, and oxalate, increasing the risk of kidney stone due to their high purine content, which promotes uric acid production during metabolism (41, 42, 43).

Additionally, healthy dietary patterns, assessed by the HEI-2020, show a significant negative correlation with biological aging indicators such as KDMAge and PhenoAge, suggesting that healthier diets may reduce the risk of kidney stones by slowing biological aging processes. This effect is achieved through mechanisms such as reduced oxidative stress and inflammation, improved metabolic pathways, and better management of metabolic syndrome-related parameters such as BMI, insulin sensitivity, and serum uric acid levels (35, 36). Healthy dietary patterns also regulate the excretion of stone-forming components in urine. For instance, moderate calcium intake binds dietary oxalate in the gut, reducing its urinary excretion, while alkaline diets decrease the risk of uric acid stone formation (10, 44). Furthermore, dietary choices, such as reducing sugar-sweetened beverages and increasing fiber-rich foods, improve gut microbiota composition, thereby promoting the growth of probiotics and reducing oxalate absorption and excretion (37, 38). Collectively, these mechanisms highlight the importance of dietary habits in kidney stone prevention, highlighting the interaction of multiple pathways, including the regulation of metabolic processes, inflammation, oxidative stress, and gut microbiota, in reducing the risk of kidney stone.

The direct association between aging and kidney stone formation is less explored; however, existing literature suggests that various physiological and lifestyle changes associated with aging may significantly contribute to stone formation (45, 46, 47). As metabolic rates decline with age, not only is energy expenditure affected, but the excretion processes of metabolites such as calcium, oxalate, and uric acid are also altered. Thus, these changes may promote stone formation, particularly among the elderly (48, 49). Our comprehensive assessment of whole-body aging using various markers consistently indicates a positive correlation between biological aging and the risk of kidney stone. Specifically, the biological age markers KDMAge and PhenoAge show promise in identifying individuals with accelerated biological aging, which could aid in preventing kidney stone development.

The mediation analysis further substantiated the mediating role of biological age in the relationship between a healthy diet and the risk of kidney stone. Our findings indicate that the aging indicators KDMAge, PhenoAge, KDMAge accelerated aging, and PhenoAge accelerated aging significantly mediated the associations between a healthy dietary pattern (the AHEI, DASHI, HEI-2020, and MEDI) and the risk of kidney stones. This pivotal finding indicates that healthy dietary patterns can reduce the risk of kidney stones and their progression by modulating biological age, underscoring the significance of biological age as a physiological marker in assessing the risk of kidney stone. Previous studies have shown that dietary patterns rich in vegetables and fruits, such as the HEI-2020, may slow the aging process due to their high levels of antioxidant and anti-inflammatory agents found in these foods (50). This finding not only supports the hypothesis that dietary modifications can slow the biological aging process but also provides a scientific foundation for dietary guidelines in clinical settings. Such dietary patterns could indirectly reduce the risk of kidney stone by ameliorating metabolic disorders and optimizing mineral metabolism, encouraging further research into how biological age influences kidney stone formation and progression and facilitating the development of personalized and targeted preventive strategies.

This study leveraged the extensive dataset provided by the NHANES to thoroughly examine the interplay between healthy dietary patterns, biological age, and kidney stone incidence. However, several limitations warrant mention. First, the use of self-reported data for kidney stones may introduce potential biases, such as memory inaccuracies, with no medical records available for validation, which could compromise the reliability of the results. Second, while efforts were made to adjust for known confounders, residual and unmeasured confounding factors might still have influenced the findings. Additionally, measurement errors, particularly in responses to the food frequency questionnaire, could have impacted study outcomes. Data on biological aging indicators were only collected at baseline, limiting our ability to analyze their temporal dynamics and effects on the risk of kidney stone, thus constraining our understanding of the influence of fluctuations in biological age. Finally, the cross-sectional nature of this study restricts our capacity to establish causality, as it captures data at a single point rather than over time, necessitating further validation through prospective or interventional studies.

## 5 Conclusion

Our analysis revealed a strong negative association between healthy dietary scores and kidney stone prevalence, while biological aging was significantly associated with an increased risk of kidney stones. Notably, the biological aging markers KDMAge, PhenoAge, KDMAge accelerated aging, and PhenoAge accelerated aging served as significant mediators in this association. These findings suggest that maintaining healthy dietary patterns may help reduce the prevalence of kidney stones and may delay their onset by decelerating biological aging. General dietary suggestions include increased fluid intake, appropriate calcium consumption, reduced high-oxalate foods, controlled animal protein intake, increased dietary fiber, and limited salt and sugar. For elderly individuals, dietary adjustments should be made gradually, with a focus on maintaining hydration and consuming easily digestible foods. High-risk groups require personalized dietary plans, strict limitations on high-oxalate foods, weight management, and regular monitoring of urine and blood tests. This study highlights the importance of dietary management in controlling biological age, thereby providing a strategic approach to the prevention and management of kidney stones.

## Data Availability

The original contributions presented in the study are included in the article/Supplementary material, further inquiries can be directed to the corresponding authors.

## References

[1] Singh P Harris PC Sas DJ Lieske JC. The genetics of kidney stone disease and nephrocalcinosis. Nat Rev Nephrol. (2022) 18:224–40. doi: 10.1038/s41581-021-00513-4 34907378

[2] Geraghty RM Cook P Walker V Somani BK. Evaluation of the economic burden of kidney stone disease in the UK: a retrospective cohort study with a mean follow-up of 19 years. BJU Int. (2020) 125:586–94. doi: 10.1111/bju.14991 31916369

[3] Crivelli JJ Maalouf NM Paiste HJ Wood KD Hughes AE Oates GR. Disparities in kidney stone disease: a scoping review. J Urol. (2021) 206:517–25. doi: 10.1097/JU.0000000000001846, PMID: 33904797 PMC8355087

[4] Scales CD Jr Smith AC Hanley JM Saigal CS. Prevalence of kidney stones in the United States. Eur Urol. (2012) 62:160–5. doi: 10.1016/j.eururo.2012.03.052 22498635 PMC3362665

[5] Khan SR Pearle MS Robertson WG Gambaro G Canales BK Doizi S. Kidney stones. Nat Rev Dis Primers. (2016) 2:16008. doi: 10.1038/nrdp.2016.8, PMID: 27188687 PMC5685519

[6] Thongprayoon C Krambeck AE Rule AD. Determining the true burden of kidney stone disease. Nat Rev Nephrol. (2020) 16:736–46. doi: 10.1038/s41581-020-0320-7, PMID: 32753740

[7] Watson G Payne SR Kunitsky K Natchagande G Mabedi C Scotland KB. Stone disease in low- and middle-income countries: could augmented reality have a role in its management? BJU Int. (2022) 130:400–7. doi: 10.1111/bju.15877 35993671

[8] Siener R. Nutrition and kidney stone disease. Nutrients. (2021) 13:1917. doi: 10.3390/nu13061917, PMID: 34204863 PMC8229448

[9] Coe FL Worcester EM Evan AP. Idiopathic hypercalciuria and formation of calcium renal stones. Nat Rev Nephrol. (2016) 12:519–33. doi: 10.1038/nrneph.2016.101 27452364 PMC5837277

[10] Ferraro PM Bargagli M Trinchieri A Gambaro G. Risk of kidney stones: influence of dietary factors, dietary patterns, and vegetarian-vegan diets. Nutrients. (2020) 12:779. doi: 10.3390/nu12030779, PMID: 32183500 PMC7146511

[11] Ticinesi A Nouvenne A Borghi L Meschi T. Water and other fluids in nephrolithiasis: state of the art and future challenges. Crit Rev Food Sci Nutr. (2017) 57:963–74. doi: 10.1080/10408398.2014.964355 25975220

[12] Peerapen P Thongboonkerd V. Kidney stone prevention. Adv Nutr. (2023) 14:555–69. doi: 10.1016/j.advnut.2023.03.002 36906146 PMC10201681

[13] Moe OW. Kidney stones: pathophysiology and medical management. Lancet. (2006) 367:333–44. doi: 10.1016/S0140-6736(06)68071-9 16443041

[14] Alexander RT Fuster DG Dimke H. Mechanisms underlying calcium nephrolithiasis. Annu Rev Physiol. (2022) 84:559–83. doi: 10.1146/annurev-physiol-052521-121822, PMID: 34699268

[15] Wang J Chen S Zhao J Liang J Gao X Gao Q. Association between nutrient patterns and hyperuricemia: mediation analysis involving obesity indicators in the NHANES. BMC Public Health. (2022) 22:1981. doi: 10.1186/s12889-022-14357-5 36307786 PMC9617335

[16] Tian T Zhang J Xie W Ni Y Fang X Liu M. Dietary quality and relationships with metabolic dysfunction-associated fatty liver disease (MAFLD) among United States adults, results from NHANES 2017-2018. Nutrients. (2022) 14:4505. doi: 10.3390/nu14214505, PMID: 36364767 PMC9659246

[17] Wollin DA Skolarikos A Preminger GM. Obesity and metabolic stone disease. Curr Opin Urol. (2017) 27:422–7. doi: 10.1097/MOU.0000000000000427, PMID: 28650866

[18] Aune D Mahamat-Saleh Y Norat T Riboli E. Body fatness, diabetes, physical activity and risk of kidney stones: a systematic review and meta-analysis of cohort studies. Eur J Epidemiol. (2018) 33:1033–47. doi: 10.1007/s10654-018-0426-4, PMID: 30066054 PMC6208979

[19] Zisman AL Evan AP Coe FL Worcester EM. Do kidney stone formers have a kidney disease? Kidney Int. (2015) 88:1240–9. doi: 10.1038/ki.2015.254, PMID: 26376133 PMC4675687

[20] Gao X Geng T Jiang M Huang N Zheng Y Belsky DW. Accelerated biological aging and risk of depression and anxiety: evidence from 424,299 UK biobank participants. Nat Commun. (2023) 14:2277. doi: 10.1038/s41467-023-38013-7 37080981 PMC10119095

[21] Chen L Wu B Mo L Chen H Zhao Y Tan T. Associations between biological ageing and the risk of, genetic susceptibility to, and life expectancy associated with rheumatoid arthritis: a secondary analysis of two observational studies. Lancet Healthy Longev. (2024) 5:e45–55. doi: 10.1016/S2666-7568(23)00220-9, PMID: 38081205

[22] Luo S Wong ICK Chui CSL Zheng J Huang Y Schooling CM. Effects of putative metformin targets on phenotypic age and leukocyte telomere length: a mendelian randomisation study using data from the UK biobank. Lancet Healthy Longev. (2023) 4:e337–44. doi: 10.1016/S2666-7568(23)00085-5 37421961

[23] Gao X Huang N Guo X Huang T. Role of sleep quality in the acceleration of biological aging and its potential for preventive interaction on air pollution insults: findings from the UK biobank cohort. Aging Cell. (2022) 21:e13610. doi: 10.1111/acel.13610 35421261 PMC9124313

[24] Belsky DW Moffitt TE Cohen AA Corcoran DL Levine ME Prinz JA. Eleven telomere, epigenetic clock, and biomarker-composite quantifications of biological aging: do they measure the same thing? Am J Epidemiol. (2018) 187:1220–30. doi: 10.1093/aje/kwx346 29149257 PMC6248475

[25] Li X Ploner A Wang Y Magnusson PK Reynolds C Finkel D. Longitudinal trajectories, correlations and mortality associations of nine biological ages across 20-years follow-up. eLife. (2020) 9:1507. doi: 10.7554/eLife.51507, PMID: 32041686 PMC7012595

[26] Tseng TY Preminger GM. Kidney stones. BMJ Clin Evid. (2011) 2011:2003. PMC3275105 22075544

[27] Kwon D Belsky DW. A toolkit for quantification of biological age from blood chemistry and organ function test data: BioAge. Geroscience. (2021) 43:2795–808. doi: 10.1007/s11357-021-00480-5 34725754 PMC8602613

[28] Levine ME Lu AT Quach A Chen BH Assimes TL Bandinelli S. An epigenetic biomarker of aging for lifespan and healthspan. Aging. (2018) 10:573–91. doi: 10.18632/aging.101414, PMID: 29676998 PMC5940111

[29] Cohen AA. Complex systems dynamics in aging: new evidence, continuing questions. Biogerontology. (2016) 17:205–20. doi: 10.1007/s10522-015-9584-x, PMID: 25991473 PMC4723638

[30] Chiuve SE Fung TT Rimm EB Hu FB McCullough ML Wang M. Alternative dietary indices both strongly predict risk of chronic disease. J Nutr. (2012) 142:1009–18. doi: 10.3945/jn.111.157222, PMID: 22513989 PMC3738221

[31] Rai SK Fung TT Lu N Keller SF Curhan GC Choi HK. The dietary approaches to stop hypertension (DASH) diet, Western diet, and risk of gout in men: prospective cohort study. BMJ. (2017) 357:j1794. doi: 10.1136/bmj.j1794 28487277 PMC5423545

[32] Shams-White MM Pannucci TE Lerman JL Herrick KA Zimmer M Meyers Mathieu K. Healthy Eating Index-2020: Review and Update Process to Reflect the Dietary Guidelines for Americans,2020-2025. J Acad Nutr Diet. (2023) 123:1280–8. doi: 10.1016/j.jand.2023.05.015 37201748 PMC10524328

[33] Sofi F Abbate R Gensini GF Casini A. Accruing evidence on benefits of adherence to the Mediterranean diet on health: an updated systematic review and meta-analysis. Am J Clin Nutr. (2010) 92:1189–96. doi: 10.3945/ajcn.2010.29673 20810976

[34] VanderWeele TJ. Explanation in causal inference: developments in mediation and interaction. Int J Epidemiol. (2016) 45:1904–8. doi: 10.1093/ije/dyw277 27864406 PMC6373498

[35] Goldfarb DS. Empiric therapy for kidney stones. Urolithiasis. (2019) 47:107–13. doi: 10.1007/s00240-018-1090-6, PMID: 30478476 PMC6361718

[36] Jian Z Wang M Jin X Li H Wang K. Diet-derived antioxidants and risk of kidney stone disease: results from the NHANES 2007-2018 and Mendelian randomization study. Front Nutr. (2021) 8:738302. doi: 10.3389/fnut.2021.738302 34993217 PMC8724258

[37] Pearle MS Goldfarb DS Assimos DG Curhan G Denu-Ciocca CJ Matlaga BR. Medical management of kidney stones: AUA guideline. J Urol. (2014) 192:316–24. doi: 10.1016/j.juro.2014.05.006 24857648

[38] Cheng JW Wagner H Asplin JR Hodgkin G Schlaifer A Fargusson M. The effect of lemonade and diet lemonade upon urinary parameters affecting calcium urinary stone formation. J Endourol. (2019) 33:160–6. doi: 10.1089/end.2018.0623 30585747

[39] Haleblian GE Leitao VA Pierre SA Robinson MR Albala DM Ribeiro AA. Assessment of citrate concentrations in citrus fruit-based juices and beverages: implications for management of hypocitraturic nephrolithiasis. J Endourol. (2008) 22:1359–66. doi: 10.1089/end.2008.0069, PMID: 18578663

[40] Trinchieri A Lizzano R Marchesotti F Zanetti G. Effect of potential renal acid load of foods on urinary citrate excretion in calcium renal stone formers. Urol Res. (2006) 34:1–7. doi: 10.1007/s00240-005-0001-9 16425021

[41] Perinpam M Ware EB Smith JA Turner ST Kardia SL Lieske JC. Effect of demographics on excretion of key urinary factors related to kidney stone risk. Urology. (2015) 86:690–6. doi: 10.1016/j.urology.2015.07.012, PMID: 26206452 PMC4592816

[42] Dongre AR Rajalakshmi M Deshmukh PR Thirunavukarasu MR Kumar R. Risk factors for kidney stones in rural Puducherry: case-control study. J Clin Diagn Res. (2017) 11:Lc01-lc05. doi: 10.7860/JCDR/2017/29465.10561 PMC5713762 29207740

[43] Asoudeh F Talebi S Jayedi A Marx W Najafi MT Mohammadi H. Associations of Total protein or animal protein intake and animal protein sources with risk of kidney stones: a systematic review and dose-response Meta-analysis. Adv Nutr. (2022) 13:821–32. doi: 10.1093/advances/nmac013 35179185 PMC9156392

[44] Ferraro PM Taylor EN Gambaro G Curhan GC. Soda and other beverages and the risk of kidney stones. Clin J Am Soc Nephrol. (2013) 8:1389–95. doi: 10.2215/CJN.11661112, PMID: 23676355 PMC3731916

[45] Lee YC Huang SP Juan YS Huang TY Liu CC. Impact of metabolic syndrome and its components on kidney stone in aging Taiwanese males. Aging Male. (2016) 19:197–201. doi: 10.1080/13685538.2016.1174987, PMID: 27138115

[46] Ye Z Wu C Xiong Y Zhang F Luo J Xu L. Obesity, metabolic dysfunction, and risk of kidney stone disease: a national cross-sectional study. Aging Male. (2023) 26:2195932. doi: 10.1080/13685538.2023.2195932 37038659

[47] Khan J Shaw S. Risk of multiple lower and upper urinary tract problems among male older adults with type-2 diabetes: a population-based study. Aging Male. (2023) 26:2208658. doi: 10.1080/13685538.2023.2208658, PMID: 37256730

[48] Kang HW Seo SP Kwon WA Woo SH Kim WT Kim YJ. Distinct metabolic characteristics and risk of stone recurrence in patients with multiple stones at the first-time presentation. Urology. (2014) 84:274–8. doi: 10.1016/j.urology.2014.02.029, PMID: 24768010

[49] Wang K Ge J Han W Wang D Zhao Y Shen Y. Risk factors for kidney stone disease recurrence: a comprehensive meta-analysis. BMC Urol. (2022) 22:62. doi: 10.1186/s12894-022-01017-4, PMID: 35439979 PMC9017041

[50] Li J Wu Z Xin S Xu Y Wang F Liu Y. Body mass index mediates the association between four dietary indices and phenotypic age acceleration in adults: a cross-sectional study. Food Funct. (2024) 15:7828–36. doi: 10.1039/d4fo01088d 38916856

