# Supplementary material for: Healthy dietary patterns, biological aging, and kidney stones: evidence from NHANES 2007–2018
Source: Front Nutr. 2025 Mar 25;12:1538289. doi: 10.3389/fnut.2025.1538289 (PMC11975591; doi:10.3389/fnut.2025.1538289)
Supplement: Supplementary file 1 [file Table_1.docx]

Table S1. Survey-weight of basic characteristics of participants by kidney stone among U.S. Adults in NHANES 2007 – 2018

| **Characteristics** | All (n=180,624,997) | Kidney stone (n=18,139,191) | Non-kidney stone (n= 162,485,806) | P-value |
| --- | --- | --- | --- | --- |
| **Age (mean ± SD)** | 48.11 ± 0.47 | 53.92 ± 0.7 | 47.46 ± 0.49 | <0.0001 |
| **Gender (%) (mean ± SD)** |  |  |  | <0.0001 |
| Male | 47.13 ± 0.67 | 53.98 ± 2.90 | 46.37 ± 0.77 |  |
| Female | 52.87 ± 0.67 | 46.02 ± 2.88 | 53.63 ± 0.77 |  |
| **Race (%) (mean ± SD)** |  |  |  | <0.0001 |
| Hispanic | 13.41 ± 1.77 | 10.24 ± 1.88 | 13.77 ± 1.80 |  |
| Non-Hispanic white | 68.29 ± 2.82 | 78.40 ± 3.02 | 67.16 ± 2.85 |  |
| Non-Hispanic black | 11.11 ± 1.37 | 5.97 ± 0.97 | 11.68 ± 1.43 |  |
| Other races | 7.19 ± 0.69 | 5.39 ± 1.14 | 7.39 ± 0.70 |  |
| **Education (%) (mean ± SD)** |  |  |  | 0.7601 |
| Less than high school education | 14.54 ± 1.08 | 14.74 ± 1.63 | 14.52 ± 1.10 |  |
| High school graduate or higher | 85.46 ± 1.16 | 85.26 ± 1.80 | 85.48 ± 1.17 |  |
| **Marital status (%) (mean ± SD)** |  |  |  | <0.0001 |
| Not married | 43.41 ± 1.44 | 36.08 ± 2.67 | 44.23 ± 1.50 |  |
| Married or living with a partner | 56.59 ± 1.46 | 63.92 ± 2.75 | 55.77 ± 1.52 |  |
| **Poverty income ratio (mean ± SD)** | 3.04 ± 0.07 | 3.07 ± 0.12 | 3.04 ± 0.08 | 0.5477 |
| **Obesity (%) (mean ± SD)** & | 38.44 ± 1.15 | 47.55 ± 2.59 | 37.42 ± 1.17 | <0.0001 |
| **Smoking (%) (mean ± SD)** ^ | 43.78 ± 1.26 | 48.30 ± 3.03 | 43.28 ± 1.28 | 0.001 |
| **Alcohol use (%) (mean ± SD)** ^^ | 73.28 ± 1.4 | 70.44 ± 3.34 | 73.60 ± 1.37 | 0.0308 |
| **Diabetes (%) (mean ± SD)** * | 15.09 ± 0.66 | 26.69 ± 2.34 | 13.81 ± 0.66 | <0.0001 |
| **High blood pressure (%) (mean ± SD)** ** | 48.5 ± 1.19 | 61.24 ± 2.84 | 47.06 ± 1.20 | <0.0001 |
| **Food insecurity (%) (mean ± SD)** | 6.87 ± 0.58 | 8.02 ± 1.34 | 6.74 ± 0.57 | 0.0462 |
| **Water Consumption (g) (mean ± SD)** | 1109.53 ± 28.79 | 1072.52 ± 53.54 | 1113.66 ± 29.36 | 0.1179 |
| **Serum Calcium (mg/dL) (mean ± SD)** | 9.39 ± 0.01 | 9.38 ± 0.03 | 9.39 ± 0.01 | 0.2056 |
| **Sitting Time (%) (mean ± SD)** |  |  |  | 0.3653 |
| <240 min/day | 36.71 ± 1.1 | 35.51 ± 2.66 | 36.85 ± 1.13 |  |
| 240-360 min/day | 22.17 ± 0.67 | 21.84 ± 2.09 | 22.20 ± 0.71 |  |
| 360-480 min/day | 18.05 ± 0.83 | 17.65 ± 2.35 | 18.09 ± 0.83 |  |
| ≥480 min/day | 23.07 ± 0.99 | 25.00 ± 2.14 | 22.86 ± 1.01 |  |
| **Dietary score (mean ± SD)** |  |  |  |  |
| AEHI | 39.22 ± 0.42 | 38.28 ± 0.68 | 39.32 ± 0.41 | 0.0006 |
| DASHI | 3.52 ± 0.03 | 3.39 ± 0.07 | 3.54 ± 0.03 | <0.0001 |
| HEI2020 | 51.60 ± 0.38 | 50.16 ± 0.68 | 51.76 ± 0.38 | <0.0001 |
| MEDI | 3.60 ± 0.04 | 3.46 ± 0.07 | 3.61 ± 0.03 | <0.0001 |
| **Indicators of aging** $ |  |  |  |  |
| KDMAge, years (mean ± SD) | 45.87 ± 0.53 | 52.08 ± 0.87 | 45.14 ± 0.53 | <0.0001 |
| PhenoAge, years (mean ± SD) | 45.59 ± 0.61 | 53.38 ± 0.98 | 44.69 ± 0.63 | <0.0001 |
| KDMAge, accelerated ageing (mean ± SD) | -2.35 ± 0.19 | -2.12 ± 0.35 | -2.38 ± 0.19 | 0.1511 |
| PhenoAge, accelerated ageing (mean ± SD) | -2.57 ± 0.21 | -0.77 ± 0.42 | -2.78 ± 0.21 | <0.0001 |
| KDMAge accelerated ageing (%) (mean ± SD) | 32.59 ± 1.30 | 34.26 ± 2.92 | 32.40 ± 1.37 | 0.2346 |
| PhenoAge accelerated ageing (%) (mean ± SD) | 27.21 ± 1.32 | 37.80 ± 2.85 | 25.98 ± 1.37 | <0.0001 |
| Homeostatic dysregulation (mean ± SD) | 2.05 ± 0.02 | 2.26 ± 0.07 | 2.03 ± 0.03 | <0.0001 |

Data are presented as mean or (%); sampling weights were applied for calculation of demographic descriptive statistics. If it is a continuous variable, the Kruskal–Wallis rank sum test was used to determine it. The p-value for continuous variables with a theoretical value of <10 was determined using Fisher’s exact probability test. With regard to categorical data, the p-value was calculated using weighted chi-square. Values are presented as means + SD or %. SD, standards deviation.

& Body mass index of 30 kg/m^2^ was considered obesity.

^ Smoking participants were defined as smoked at least 100 cigarettes in life.

^^ Alcohol participants were defined as had at least 12 alcohol drinks 1 year.

* Diagnosed diabetes was defined as a fasting glucose level of 7 mmol/L (126 mg/dL), a hemoglobin A1c level of 6.5%, or a diagnosed diabetes.

** Systolic blood pressure of 130 mm Hg, diastolic blood pressure of 80 mm Hg, or the use of antihypertensive drugs was considered high blood pressure.

$ Accelerated biological ageing was defined as a KDMAge acceleration or PhenoAge acceleration greater than 0, whereas non-accelerated ageing was defined as a KDMAge acceleration or PhenoAge acceleration less than or equal to 0.

Table S2

Multiple linear regression and logistic regression associations of healthy dietary score and aging indicators

| Exposure* | AHEI-Log2 |  | DASHI-Log2 |  | HEI2020-Log2 |  | MEDI-Log2 |  |
| --- | --- | --- | --- | --- | --- | --- | --- | --- |
|  | β/OR (95%CI) | p-value | β/OR (95%CI) | p-value | β/OR (95%CI) | p-value | β/OR (95%CI) | p-value |
| KDMAge | -0.94 (-1.19, -0.70) | <0.0001 | -0.82 (-1.04, -0.60) | <0.0001 | -1.30 (-1.64, -0.96) | <0.0001 | -0.70 (-0.93, -0.46) | <0.0001 |
| KDMAge -Accelerated ageing | 0.76 (0.69, 0.84) | <0.0001 | 0.78 (0.71, 0.85) | <0.0001 | 0.66 (0.58, 0.76) | <0.0001 | 0.84 (0.76, 0.92) | 0.0003 |
| PhenoAge | -1.51 (-1.78, -1.25) | <0.0001 | -1.12 (-1.35, -0.90) | <0.0001 | -2.10 (-2.44, -1.75) | <0.0001 | -0.94 (-1.18, -0.69) | <0.0001 |
| PhenoAge -Accelerated ageing | 0.60 (0.54, 0.67) | <0.0001 | 0.48 (0.41, 0.55) | <0.0001 | 0.71 (0.64, 0.79) | <0.0001 | 0.67 (0.62, 0.74) | <0.0001 |
| Homeostatic dysregulation | -0.09 (-0.13, -0.06) | <0.0001 | -0.04 (-0.07, -0.01) | 0.0309 | -0.11 (-0.16, -0.06) | <0.0001 | -0.06 (-0.09, -0.02) | 0.006 |

In this analysis, we adjusted for age, sex, race, marital status, education level, poverty income ratio, obesity, smoking, alcohol use, diabetes, high blood pressure, food insecurity, water consumption, serum calcium, and sitting time.

*For the KDMAge -Accelerated ageing and PhenoAge -Accelerated ageing, we used the logistic regression to analyze the association with healthy dietary score. For the KDMAge, PhenoAge and Homeostatic dysregulation, we used the linear regression to analyze the association with healthy dietary score.

Table S3. The mediation effects of aging indicators and accelerated aging on the association of healthy dietary score with kidney stone in adults

| Exposure | Mediators | Indirect effects | 95%CI | P-value | Direct effects | 95%CI | P-value | Total effects | 95%CI | P-value | Mediated proportion (%) | p value |
| --- | --- | --- | --- | --- | --- | --- | --- | --- | --- | --- | --- | --- |
| **AHEI** | **KDMAge** | **-0.000632** | **(-0.001203,-0.000097)** | **0.026** | **-0.008402** | **(-0.016302,-0.000542)** | **0.026** | **-0.009034** | **(-0.016667,-0.001244)** | **0.014** | **6.9992** | **0.04** |
|  | **PhenoAge** | **-0.000994** | **(-0.001856,-0.000206)** | **0.016** | **-0.009331** | **(-0.017479,-0.001091)** | **0.03** | **-0.010325** | **(-0.018460,-0.002264)** | **0.016** | **9.6309** | **0.032** |
|  | Homeostatic dysregulation | -0.000168 | (-0.000556,0.000198) | 0.404 | -0.008404 | (-0.016172,-0.000910) | 0.034 | -0.008572 | (-0.016250,-0.001060) | 0.024 | 1.9594 | 0.424 |
|  | **KDMAge accelerated aging** | **-0.000491** | **(-0.001103,-0.000110)** | **0.008** | **-0.00858** | **(-0.016534,-0.000990)** | **0.024** | **-0.009072** | **(-0.017123,-0.001555)** | **0.016** | **5.4167** | **0.024** |
|  | **PhenoAge accelerated aging** | **-0.001055** | **(-0.001972,-0.000292)** | **0.002** | **-0.009075** | **(-0.016776,-0.001129)** | **0.036** | **-0.01013** | **(-0.017888,-0.002132)** | **0.016** | **10.4156** | **0.018** |
| **DASH** | **KDMAge** | **-0.000629** | **(-0.001207,-0.000055)** | **0.036** | **-0.018968** | **(-0.027417,-0.010993)** | **<0.0001** | **-0.019597** | **(-0.028055,-0.011566)** | **<0.0001** | **3.2074** | **0.036** |
|  | **PhenoAge** | **-0.000873** | **(-0.001667,-0.000139)** | **0.022** | **-0.018119** | **(-0.026506,-0.009565)** | **<0.0001** | **-0.018992** | **(-0.027299,-0.010361)** | **<0.0001** | **4.5988** | **0.022** |
|  | Homeostatic dysregulation | -0.00008 | (-0.000312,0.000137) | 0.458 | -0.019485 | (-0.027980,-0.011464) | <0.0001 | -0.019565 | (-0.027998,-0.011537) | <0.0001 | 0.4075 | 0.458 |
|  | **KDMAge accelerated aging** | **-0.000412** | **(-0.001077,-0.000058)** | **0.026** | **-0.018852** | **(-0.027300,-0.010882)** | **<0.0001** | **-0.019264** | **(-0.027859,-0.011258)** | **<0.0001** | **2.1393** | **0.026** |
|  | **PhenoAge accelerated aging** | **-0.000894** | **(-0.001770,-0.000235)** | **0.002** | **-0.017963** | **(-0.026308,-0.009246)** | **<0.0001** | **-0.018857** | **(-0.027085,-0.010088)** | **<0.0001** | **4.7394** | **0.002** |
| **HEI2020** | **KDMAge** | **-0.000636** | **(-0.001215,-0.000025)** | **0.038** | **-0.020741** | **(-0.029231,-0.013037)** | **<0.0001** | **-0.021377** | **(-0.029918,-0.013759)** | **<0.0001** | **2.9759** | **0.038** |
|  | **PhenoAge** | **-0.000979** | **(-0.001924,-0.000096)** | **0.034** | **-0.020679** | **(-0.028845,-0.011710)** | **<0.0001** | **-0.021658** | **(-0.029795,-0.012506)** | **<0.0001** | **4.5194** | **0.034** |
|  | Homeostatic dysregulation | -0.000121 | (-0.000518,0.000256) | 0.524 | -0.02136 | (-0.029943,-0.013650) | <0.0001 | -0.02148 | (-0.030085,-0.013746) | <0.0001 | 0.5619 | 0.524 |
|  | **KDMAge accelerated aging** | **-0.000502** | **(-0.001131,-0.000040)** | **0.036** | **-0.0208** | **(-0.029639,-0.012886)** | **<0.0001** | **-0.021302** | **(-0.030042,-0.013558)** | **<0.0001** | **2.3556** | **0.036** |
|  | **PhenoAge accelerated aging** | **-0.001039** | **(-0.002028,-0.000214)** | **0.01** | **-0.020717** | **(-0.028833,-0.011686)** | **<0.0001** | **-0.021756** | **(-0.029908,-0.012560)** | **<0.0001** | **4.775** | **0.01** |
| **MEDI** | **KDMAge** | **-0.00035** | **(-0.000681,-0.000044)** | **0.028** | **-0.011253** | **(-0.016960,-0.005699)** | **<0.0001** | **-0.011604** | **(-0.017375,-0.006075)** | **<0.0001** | **3.0202** | **0.028** |
|  | **PhenoAge** | **-0.00047** | **(-0.000898,-0.000086)** | **0.02** | **-0.011678** | **(-0.017137,-0.006209)** | **<0.0001** | **-0.012148** | **(-0.017623,-0.006850)** | **<0.0001** | **3.8678** | **0.02** |
|  | Homeostatic dysregulation | -0.000069 | (-0.000262,0.000129) | 0.476 | -0.01168 | (-0.017664,-0.006259) | <0.0001 | -0.011748 | (-0.017737,-0.006297) | <0.0001 | 0.5843 | 0.476 |
|  | **KDMAge accelerated aging** | **-0.000185** | **(-0.000650,-0.000004)** | **0.05** | **-0.011792** | **(-0.017811,-0.006136)** | **<0.0001** | **-0.011977** | **(-0.018006,-0.006579)** | **<0.0001** | **1.5439** | **0.05** |
|  | **PhenoAge accelerated aging** | **-0.000433** | **(-0.001026,-0.000114)** | **0.006** | **-0.012104** | **(-0.017493,-0.006492)** | **<0.0001** | **-0.012537** | **(-0.018167,-0.007173)** | **<0.0001** | **3.453** | **0.006** |

Accelerated biological ageing was defined as a KDMAge acceleration or PhenoAge acceleration greater than 0, whereas non-accelerated ageing was defined as a KDMAge acceleration or PhenoAge acceleration less than or equal to 0.

In this analysis, we adjusted for age, sex, race, marital status, education level, poverty income ratio, obesity, smoking, alcohol use, diabetes, high blood pressure, food insecurity, water consumption, serum calcium, and sitting time.

Table S4. Association between healthy dietary scores and risk of prevalent kidney stone (Complete case)

|  | Model 1 | | Model 2 | | Model 3 | |
| --- | --- | --- | --- | --- | --- | --- |
| Dietary Score | OR (95%CI) | P-value | OR (95%CI) | P-value | OR (95%CI) | P-value |
| AHEI-Continuous (Log2) | 0.89 (0.82, 0.97) | 0.0091 | 0.75 (0.68, 0.82) | <0.0001 | 0.80 (0.72, 0.88) | <0.0001 |
| Q1 | Reference |  | Reference |  | Reference |  |
| Q2 | 1.00 (0.89, 1.12) | 0.9977 | 0.91 (0.81, 1.02) | 0.1165 | 0.92 (0.82, 1.04) | 0.188 |
| Q3 | 1.00 (0.89, 1.12) | 0.9769 | 0.84 (0.75, 0.95) | 0.0044 | 0.87 (0.77, 0.98) | 0.0212 |
| Q4 | 0.88 (0.78, 0.99) | 0.0281 | 0.71 (0.63, 0.80) | <0.0001 | 0.77 (0.68, 0.88) | <0.0001 |
| P for trend |  | 0.039 |  | <0.001 |  | <0.001 |
| DASHI-Continuous (Log2) | 0.80 (0.74, 0.86) | <0.0001 | 0.77 (0.71, 0.83) | <0.0001 | 0.80 (0.74, 0.87) | <0.0001 |
| Q1 | Reference |  | Reference |  | Reference |  |
| Q2 | 0.98 (0.88, 1.09) | 0.7175 | 0.96 (0.86, 1.08) | 0.5173 | 0.97 (0.87, 1.09) | 0.6572 |
| Q3 | 0.86 (0.77, 0.96) | 0.0081 | 0.83 (0.74, 0.93) | 0.0019 | 0.87 (0.77, 0.98) | 0.0173 |
| Q4 | 0.72 (0.64, 0.81) | <0.0001 | 0.67 (0.59, 0.76) | <0.0001 | 0.71 (0.63, 0.81) | <0.0001 |
| P for trend |  | <0.001 |  | <0.001 |  | <0.001 |
| HEI2020-Continous (Log2) | 0.74 (0.66, 0.83) | <0.0001 | 0.60 (0.54, 0.68) | <0.0001 | 0.66 (0.58, 0.75) | <0.0001 |
| Q1 | Reference |  | Reference |  | Reference |  |
| Q2 | 0.98 (0.88, 1.09) | 0.694 | 0.92 (0.82, 1.03) | 0.1354 | 0.94 (0.84, 1.05) | 0.2715 |
| Q3 | 0.88 (0.78, 0.98) | 0.0242 | 0.78 (0.70, 0.88) | <0.0001 | 0.83 (0.73, 0.93) | 0.0015 |
| Q4 | 0.78 (0.69, 0.87) | <0.0001 | 0.64 (0.57, 0.72) | <0.0001 | 0.69 (0.61, 0.78) | <0.0001 |
| P for trend |  | <0.001 |  | <0.001 |  | <0.001 |
| MEDI-Continuous (Log2) | 0.80 (0.74, 0.87) | <0.0001 | 0.77 (0.70, 0.83) | <0.0001 | 0.79 (0.72, 0.86) | <0.0001 |
| Q1 | Reference |  | Reference |  | Reference |  |
| Q2 | 1.00 (0.88, 1.14) | 0.9739 | 0.96 (0.84, 1.09) | 0.5085 | 0.96 (0.84, 1.09) | 0.5293 |
| Q3 | 0.93 (0.82, 1.05) | 0.2573 | 0.88 (0.77, 1.00) | 0.0449 | 0.88 (0.77, 0.99) | 0.0404 |
| Q4 | 0.79 (0.71, 0.88) | <0.0001 | 0.74 (0.66, 0.83) | <0.0001 | 0.76 (0.68, 0.85) | <0.0001 |
| P for trend |  | <0.001 |  | <0.001 |  | <0.001 |

Model 1 was adjusted for none. Model 2 was adjusted for age, sex, race, marital status, education level, and poverty income ratio. Model 3 was adjusted for age, sex, race, marital status, education level, poverty income ratio, obesity, smoking, alcohol use, diabetes, high blood pressure, food insecurity, water consumption, serum calcium, and sitting time.

Table S5. Multiple logistic regression of associations of indicators of aging with kidney stone in adults (Complete case)

| Exposure |  | Model 1 | | Model 2 | |
| --- | --- | --- | --- | --- | --- |
|  |  | Odds ratio (95% CI) | p value | Odds ratio (95% CI) | p value |
| KDMAge | Continuous (Log2) | 3.20 (2.46, 4.18) | <0.0001 | 2.21 (1.66, 2.94) | <0.0001 |
|  | Q1 | Reference |  | Reference |  |
|  | Q2 | 1.97 (1.59, 2.45) | <0.0001 | 1.64 (1.31, 2.05) | <0.0001 |
|  | Q3 | 2.59 (1.94, 3.46) | <0.0001 | 1.83 (1.35, 2.48) | 0.0001 |
|  | Q4 | 2.95 (2.04, 4.28) | <0.0001 | 1.83 (1.24, 2.70) | 0.0025 |
|  | Non-accelerated ageing | Reference |  | Reference |  |
|  | Accelerated ageing | 1.37 (1.23, 1.54) | <0.0001 | 1.19 (1.06, 1.34) | 0.004 |
| PhenoAge | Continuous (Log2) | 3.21 (2.54, 4.07) | <0.0001 | 2.24 (1.74, 2.88) | <0.0001 |
|  | Q1 | Reference |  | Reference |  |
|  | Q2 | 2.06 (1.65, 2.56) | <0.0001 | 1.67 (1.34, 2.09) | <0.0001 |
|  | Q3 | 2.82 (2.10, 3.78) | <0.0001 | 1.85 (1.37, 2.51) | <0.0001 |
|  | Q4 | 3.35 (2.29, 4.90) | <0.0001 | 1.81 (1.21, 2.69) | 0.0038 |
|  | Non-accelerated ageing | Reference |  | Reference |  |
|  | Accelerated ageing | 1.51 (1.35, 1.68) | <0.0001 | 1.26 (1.12, 1.43) | 0.0001 |
| Homeostatic dysregulation | Continuous (Log2) | 1.33 (1.20, 1.48) | <0.0001 | 1.07 (0.95, 1.20) | 0.2679 |
|  | Q1 | Reference |  | Reference |  |
|  | Q2 | 1.20 (1.02, 1.42) | 0.0301 | 1.12 (0.95, 1.33) | 0.18 |
|  | Q3 | 1.36 (1.15, 1.61) | 0.0004 | 1.13 (0.95, 1.35) | 0.169 |
|  | Q4 | 1.57 (1.32, 1.88) | <0.0001 | 1.16 (0.95, 1.41) | 0.1459 |

Accelerated biological ageing was defined as a KDMAge acceleration or PhenoAge acceleration greater than 0, whereas non-accelerated ageing was defined as a KDMAge acceleration or PhenoAge acceleration less than or equal to 0. Model 1 was adjusted for age, sex, race, marital status, education level, and poverty income ratio. Model 2 was adjusted for age, sex, race, marital status, education level, poverty income ratio, obesity, smoking, alcohol use, diabetes, high blood pressure, food insecurity, water consumption, serum calcium, and sitting time.

Table S6. Multiple logistic regression associations of indicators of aging with kidney stone in adults (Complete case)

|  | AHEI-Log2 |  | DASHI-Log2 |  | HEI2020-Log2 |  | MEDI-Log2 |  |
| --- | --- | --- | --- | --- | --- | --- | --- | --- |
|  | β/OR (95%CI) | p-value | β/OR (95%CI) | p-value | β/OR (95%CI) | p-value | β/OR (95%CI) | p-value |
| KDMAge | -0.99 (-1.20, -0.79) | <0.0001 | -0.81 (-0.99, -0.62) | <0.0001 | -1.34 (-1.62, -1.06) | <0.0001 | -0.72 (-0.92, -0.52) | <0.0001 |
| KDMAge -Accelerated ageing | 0.77 (0.70, 0.83) | <0.0001 | 0.77 (0.72, 0.83) | <0.0001 | 0.67 (0.60, 0.75) | <0.0001 | 0.85 (0.78, 0.92) | <0.0001 |
| PhenoAge | -1.49 (-1.70, -1.28) | <0.0001 | -1.10 (-1.29, -0.92) | <0.0001 | -2.02 (-2.30, -1.74) | <0.0001 | -0.97 (-1.17, -0.77) | <0.0001 |
| PhenoAge -Accelerated ageing | 0.60 (0.55, 0.65) | <0.0001 | 0.68 (0.64, 0.74) | <0.0001 | 0.49 (0.44, 0.55) | <0.0001 | 0.70 (0.65, 0.76) | <0.0001 |
| Homeostatic dysregulation | -0.09 (-0.12, -0.06) | <0.0001 | -0.04 (-0.06, -0.02) | 0.0013 | -0.11 (-0.15, -0.08) | <0.0001 | -0.06 (-0.08, -0.03) | <0.0001 |

In this analysis, we adjusted for age, sex, race, marital status, education level, poverty income ratio, obesity, smoking, alcohol use, diabetes, high blood pressure, food insecurity, water consumption, serum calcium, and sitting time.

*For the KDMAge -Accelerated ageing and PhenoAge -Accelerated ageing, we used the logistic regression to analyze the association with healthy dietary score. For the KDMAge, PhenoAge and Homeostatic dysregulation, we used the linear regression to analyze the association with healthy dietary score.

Table S7. Association between healthy dietary scores and risk of prevalent kidney stone by 6-year Survey Cycle, NHANES 2007-2018

| Dietary Score | NHANES 2007-2012 | | NHANES 2013- 2018 | |
| --- | --- | --- | --- | --- |
|  | OR (95%CI) | P-value | OR (95%CI) | P-value |
| AHEI-Continuous (Log2) | 0.77 (0.66, 0.89) | 0.0005 | 0.93 (0.78, 1.11) | 0.419 |
| Q1 | Reference |  | Reference |  |
| Q2 | 0.88 (0.74, 1.06) | 0.1843 | 1.02 (0.82, 1.27) | 0.8338 |
| Q3 | 0.72 (0.60, 0.88) | 0.001 | 1.03 (0.83, 1.29) | 0.7565 |
| Q4 | 0.70 (0.58, 0.85) | 0.0004 | 0.92 (0.73, 1.17) | 0.5056 |
| P for trend |  | <0.001 |  | 0.563 |
| DASHI-Continuous (Log2) | 0.77 (0.68, 0.88) | 0.0001 | 0.87 (0.75, 1.00) | 0.0547 |
| Q1 | Reference |  | Reference |  |
| Q2 | 0.74 (0.62, 0.89) | 0.0011 | 0.97 (0.79, 1.19) | 0.7902 |
| Q3 | 0.78 (0.66, 0.94) | 0.0076 | 0.93 (0.76, 1.16) | 0.5365 |
| Q4 | 0.66 (0.54, 0.80) | <0.0001 | 0.81 (0.64, 1.02) | 0.071 |
| P for trend |  | <0.001 |  | 0.073 |
| HEI2020-Continous (Log2) | 0.59 (0.49, 0.72) | <0.0001 | 0.78 (0.63, 0.97) | 0.0269 |
| Q1 | Reference |  | Reference |  |
| Q2 | 0.88 (0.74, 1.06) | 0.175 | 0.96 (0.78, 1.19) | 0.7214 |
| Q3 | 0.72 (0.60, 0.87) | 0.0006 | 1.01 (0.82, 1.25) | 0.9343 |
| Q4 | 0.65 (0.54, 0.79) | <0.0001 | 0.75 (0.60, 0.95) | 0.0175 |
| P for trend |  | <0.001 |  | 0.043 |
| MEDI-Continuous (Log2) | 0.75 (0.65, 0.86) | <0.0001 | 0.86 (0.74, 1.01) | 0.074 |
| Q1 | Reference |  | Reference |  |
| Q2 | 0.93 (0.76, 1.14) | 0.4908 | 0.96 (0.76, 1.21) | 0.722 |
| Q3 | 0.87 (0.71, 1.06) | 0.1624 | 0.95 (0.77, 1.16) | 0.5992 |
| Q4 | 0.73 (0.61, 0.87) | 0.0004 | 0.79 (0.62, 0.99) | 0.0446 |
| P for trend |  | <0.001 |  | 0.063 |

In this analysis, we adjusted for age, sex, race, marital status, education level, poverty income ratio, obesity, smoking, alcohol use, diabetes, high blood pressure, food insecurity, water consumption, serum calcium, and sitting time.

Table S8. Multiple logistic regression associations of indicators of aging with kidney stone in adults by 6-year Survey Cycle, NHANES 2007-2018

| Exposure |  | NHANES 2007-2012 | | NHANES 2013- 2018 | |
| --- | --- | --- | --- | --- | --- |
|  |  | Odds ratio (95% CI) | p value | Odds ratio (95% CI) | p value |
| KDMAge | Continuous (Log2) | 2.47 (1.60, 3.82) | <0.0001 | 2.12 (1.18, 3.81) | 0.0116 |
|  | Q1 | Reference |  | Reference |  |
|  | Q2 | 1.55 (1.09, 2.19) | 0.0137 | 2.27 (1.41, 3.65) | 0.0008 |
|  | Q3 | 1.62 (1.01, 2.60) | 0.0449 | 2.98 (1.61, 5.52) | 0.0005 |
|  | Q4 | 1.46 (0.79, 2.70) | 0.2235 | 3.12 (1.49, 6.56) | 0.0027 |
|  | Non-accelerated ageing | Reference |  | Reference |  |
|  | Accelerated ageing | 1.20 (1.01, 1.44) | 0.0412 | 1.15 (0.91, 1.44) | 0.2513 |
| PhenoAge | Continuous (Log2) | 2.21 (1.49, 3.27) | <0.0001 | 2.24 (1.34, 3.74) | 0.002 |
|  | Q1 | Reference |  | Reference |  |
|  | Q2 | 1.50 (1.06, 2.13) | 0.0239 | 2.29 (1.41, 3.71) | 0.0008 |
|  | Q3 | 1.81 (1.12, 2.94) | 0.0163 | 2.15 (1.15, 3.99) | 0.0158 |
|  | Q4 | 1.74 (0.92, 3.28) | 0.0885 | 1.96 (0.90, 4.31) | 0.0919 |
|  | Non-accelerated ageing | Reference |  | Reference |  |
|  | Accelerated ageing | 1.22 (1.00, 1.48) | 0.0475 | 1.20 (0.95, 1.51) | 0.1329 |
| Homeostatic dysregulation | Continuous (Log2) | 1.06 (0.89, 1.26) | 0.5465 | 1.20 (0.96, 1.50) | 0.1117 |
|  | Q1 | Reference |  | Reference |  |
|  | Q2 | 1.09 (0.84, 1.40) | 0.5251 | 1.20 (0.83, 1.73) | 0.3343 |
|  | Q3 | 1.11 (0.85, 1.45) | 0.4509 | 1.41 (0.97, 2.05) | 0.0738 |
|  | Q4 | 1.17 (0.88, 1.57) | 0.2868 | 1.44 (0.96, 2.17) | 0.0782 |

In this analysis, we adjusted for age, sex, race, marital status, education level, poverty income ratio, obesity, smoking, alcohol use, diabetes, high blood pressure, food insecurity, water consumption, serum calcium, and sitting time.

For the KDMAge -Accelerated ageing and PhenoAge -Accelerated ageing, we used the logistic regression to analyze the association with healthy dietary score. For the KDMAge, PhenoAge and Homeostatic dysregulation, we used the linear regression to analyze the association with healthy dietary score.

Table S9. Specific dietary components of participants grouped by kidney stone among U.S. Adults in NHANES 2007 - 2018

| **Specific dietary components*** | All | Kidney stone | Non-kidney stone | P-value |
| --- | --- | --- | --- | --- |
| **HEI2020 (mean ± SD)** |  |  |  |  |
| Total fruit | 2.17 ± 1.78 | 2.04 ± 1.75 | 2.19 ± 1.78 | 0.005 |
| Fruit | 2.19 ± 1.92 | 2.08 ± 1.89 | 2.20 ± 1.93 | 0.033 |
| Vegetable | 3.05 ± 1.36 | 2.99 ± 1.35 | 3.05 ± 1.36 | 0.147 |
| Green bean | 1.66 ± 1.72 | 1.56 ± 1.68 | 1.67 ± 1.73 | 0.032 |
| Total Meat | 4.26 ± 0.98 | 4.22 ± 0.99 | 4.26 ± 0.98 | 0.441 |
| Seafood | 2.37 ± 1.80 | 2.33 ± 1.79 | 2.38 ± 1.80 | 0.441 |
| Whole grains | 2.68 ± 2.91 | 2.60 ± 2.88 | 2.69 ± 2.92 | 0.281 |
| Dairy | 4.65 ± 2.76 | 4.63 ± 2.70 | 4.65 ± 2.77 | 0.784 |
| Fatty Acids | 5.19 ± 2.86 | 5.00 ± 2.80 | 5.21 ± 2.87 | 0.014 |
| Sodium | 4.27 ± 2.79 | 4.28 ± 2.77 | 4.27 ± 2.80 | 0.839 |
| Added sugar | 6.98 ± 2.84 | 6.76 ± 2.96 | 7.01 ± 2.83 | 0.002 |
| Saturated Fatty Acids | 5.96 ± 2.81 | 5.63 ± 2.77 | 6.00 ± 2.81 | <0.001 |
| **AHEI (mean ± SD)** |  |  |  |  |
| Vegetable | 3.83 ± 2.55 | 3.61 ± 2.44 | 3.85 ± 2.56 | <0.001 |
| Fruit | 1.75 ± 2.09 | 1.59 ± 1.95 | 1.77 ± 2.10 | <0.001 |
| Whole grains | 2.63 ± 2.87 | 2.51 ± 2.83 | 2.64 ± 2.88 | 0.092 |
| Nuts | 4.08 ± 3.66 | 4.11 ± 3.61 | 4.08 ± 3.66 | 0.715 |
| Omega 3 fatty acid | 2.27 ± 2.27 | 2.16 ± 2.15 | 2.28 ± 2.29 | 0.08 |
| PUFA | 6.66 ± 2.24 | 6.74 ± 2.24 | 6.65 ± 2.24 | 0.165 |
| Total Meat | 5.46 ± 3.20 | 5.27 ± 3.09 | 5.49 ± 3.21 | 0.011 |
| Sodium | 4.67 ± 2.49 | 4.69 ± 2.46 | 4.67 ± 2.49 | 0.85 |
| Alcohol | 2.03 ± 2.06 | 1.97 ± 1.98 | 2.04 ± 2.06 | 0.281 |
| **DASHI (mean ± SD)** |  |  |  |  |
| Fat | 0.39 ± 0.33 | 0.36 ± 0.32 | 0.39 ± 0.33 | <0.001 |
| Saturated fat | 0.51 ± 0.26 | 0.48 ± 0.25 | 0.51 ± 0.26 | <0.001 |
| Protein | 0.41 ± 0.35 | 0.38 ± 0.35 | 0.41 ± 0.35 | 0.003 |
| Cholesterol | 0.42 ± 0.33 | 0.43 ± 0.33 | 0.42 ± 0.33 | 0.385 |
| Fiber | 0.40 ± 0.29 | 0.38 ± 0.27 | 0.41 ± 0.29 | 0.004 |
| Magnesium | 0.44 ± 0.26 | 0.43 ± 0.25 | 0.44 ± 0.26 | 0.02 |
| Calcium | 0.55 ± 0.29 | 0.55 ± 0.29 | 0.56 ± 0.29 | 0.526 |
| Sodium | 0.21 ± 0.28 | 0.21 ± 0.28 | 0.21 ± 0.28 | 0.868 |
| **MEDI (mean ± SD)** |  |  |  |  |
| Olive oil | 0.00 ± 0.01 | 0.00 ± 0.00 | 0.00 ± 0.01 | 0.45 |
| Whole fruits | 0.05 ± 0.16 | 0.04 ± 0.15 | 0.05 ± 0.17 | 0.04 |
| Vegetables | 0.15 ± 0.27 | 0.13 ± 0.26 | 0.15 ± 0.28 | 0.005 |
| Legumes | 0.21 ± 0.31 | 0.20 ± 0.31 | 0.21 ± 0.32 | 0.505 |
| Nuts | 0.25 ± 0.35 | 0.25 ± 0.35 | 0.25 ± 0.35 | 0.996 |
| Seafood | 0.13 ± 0.25 | 0.12 ± 0.24 | 0.13 ± 0.25 | 0.405 |
| Alcohol | 0.16 ± 0.31 | 0.13 ± 0.29 | 0.16 ± 0.31 | 0.003 |
| Sweets | 0.35 ± 0.37 | 0.31 ± 0.36 | 0.35 ± 0.37 | <0.001 |
| Meats | 0.85 ± 0.27 | 0.85 ± 0.27 | 0.85 ± 0.27 | 0.917 |

*HEI2020:

Total fruit: The serving size of total fruits including fruit juice, unit= cup eq.; Fruit: The serving size of Citrus, Melons, Berries + Other Intact Fruits, unit= cup eq.; Vegetable: The serving size of vegetables Total Vegetables + Legumes (Beans and Peas) in cup equivalents, unit= cup eq.; Green bean: The serving size of Dark Green Vegetables + Legumes (Beans and Peas) in cup equivalents, unit= cup eq.; Total Meat: The serving size of Total Meat, Poultry, and Seafood (including organ meats and cured meats) + Eggs + Nuts and Seeds + Soy + Legumes (Beans and Peas) in oz equivalents, unit=oz. eq., 1 cup legume = 4 oz; Seafood: The serving size of Seafood (high in n-3) + Seafood (low in n-3) + Soy + Nuts and Seeds + Legumes (Beans and Peas) in oz equivalents, unit=oz. eq., 1 cup legume = 4 oz; Whole grains: The serving size of whole grains, unit=oz. eq.; Dairy: The serving size of all dairy, unit=cup eq. Fatty Acids: The serving size of (Total Monounsaturated Fatty Acids + Total Polyunsaturated Fatty Acids)/Total Saturated Fatty Acids, unit=g; Sodium: The serving size of sodium, unit=mg; Added sugar: The serving size of added sugar, unit=tsp

AHEI:

Vegetable: The serving size of All vegetable except potatoes and legume, unit=servings/day (0.5 c of vege; 1 cup of green leafy (1 cup = 236.59 g); Fruit: The serving size of All whole fruits and no fruit juice, unit=servings/day (0.5 c of berries; 1 cup=236.59 g; 1 med fruit (1 cup = 236.59 g); Whole grains: The serving size of whole grains, unit=grams/day; Nuts: The serving size of Nuts, legumes, and vegetable protein (e.g., tofu), unit=servings/day = 1 srv=1oz (28.35 g) of nuts and legume or 1 TBLSP peanut butter (15 mL), 1 cup legume = 4 oz; Omega 3 fatty acid: The serving size of omega 3 fatty acid, unit=mg/day ( oz. = 28.35 g); PUFA: The serving size of PUFA, unit=% of energy; Polyunsaturated fatty acids (PUFAs); Total Meat: The serving size of red and processed meats, including Beef, pork, lamb, goat, veal, sausages, bacon, salami, ham, hot dog, deli meat, unit=servings/day; 1 srv= 4 oz. unprocessed meat; 1.5 oz. processed meat (1 oz. = 28.35 g); Sodium: The serving size of sodium, unit=mg/day per 2000 kcal; Alcohol: The serving size of alcohol, including Wine, beer, "light" beer, liquor, unit=drink/day (12 oz beer; 5 oz wine; 1.5 oz spirits) 1 oz = 28.35 g

DASHI:

Fat: The total fat intake, unit = g/day; Saturated fat: The saturated fat intake, unit = g/day; Protein: The protein intake, unit = g/day; Cholesterol: The cholesterol intake, unit = mg/day; Fiber: The fiber intake, unit = g/day; Magnesium: The magnesium intake, unit = mg/day; Calcium: The calcium intake, unit = mg/day; Sodium: The sodium intake, unit = mg/day.

MEDI:

Olive oil: The serving size of olive oil, unit= 10 g or 0.8 tbsp = 1 serving; Whole fruits: The serving size of All whole fruits, unit= 125 g = 1 serving (approximately 1 c); Vegetables: The serving size of All vegetables except potatoes and legumes, unit= 125 g = 1 serving (approximately 1 c of non-green leafy vege; 2 cup of green leafy vege); Legumes: The serving size of legumes, including Dried beans, lentils, peas, soups (split pea), tofu, soymilk, unit= 40 g = 1 serving (about 1.5 oz); Nuts: The serving size of nuts and seeds, including Peanuts, almonds, sunflower seeds, cashews, walnuts, unit= 25 g = 1 serving (about 1 oz); Seafood: The serving size of all fishes and seafoods, including Fresh-water and sea-water fish; preserved fish such as salted fish, canned fish; shellfish (squid, prawns, mollusks), unit=125 g = 1 serving (about 4 oz); Alcohol: The serving size of alcohol, including Wine, beer, "light" beer, liquor, unit= 14 g pure alcohol = 1 serving (12 ounces of beer, 5 ounces of wine, or 1.5 ounces of hard liquor, such as vodka or whiskey); Sweets: The serving size of all sweets, including Candy, chocolate, ice cream, cookies, cakes, pies, pastries, unit= 50 g = 1 serving (about 1.5 oz ) = 1 piece of candy, 1 chocolate bar, 1 scoop ice cream, 1 cookie, 1 cake slice, 1 pie slice, 1 pastry; Meats: The serving size of red and processed meats, including Beef, pork, lamb, goat, veal, sausages, bacon, salami, ham, hot dog, deli meat, unit= 150 g = 1 serving (about 5 oz)

Table S10. Association between specific dietary components and kidney stones

| **Specific dietary components*** | Model 1 | | Model 2 | |
| --- | --- | --- | --- | --- |
|  | OR (95%CI) | P-value | OR (95%CI) | P-value |
| **HEI2020 (Log2)** |  |  |  |  |
| Total fruit | 0.95 (0.92, 0.99) | 0.0089 | 0.97 (0.93, 1.01) | 0.1823 |
| Fruit | 0.93 (0.89, 0.98) | 0.0027 | 0.93 (0.88, 0.98) | 0.0115 |
| Vegetable | 0.96 (0.90, 1.03) | 0.2464 | 1.00 (0.93, 1.08) | 0.9206 |
| Green bean | 1.00 (0.93, 1.08) | 0.9542 | 1.00 (0.92, 1.10) | 0.9184 |
| Total Meat | 0.95 (0.85, 1.06) | 0.3585 | 0.93 (0.81, 1.07) | 0.3235 |
| Seafood | 1.00 (0.96, 1.06) | 0.8436 | 1.03 (0.96, 1.09) | 0.4379 |
| Whole grains | 0.96 (0.92, 1.02) | 0.1647 | 1.00 (0.93, 1.07) | 0.9116 |
| Dairy | 0.96 (0.92, 1.00) | 0.0558 | 0.96 (0.91, 1.02) | 0.16 |
| Fatty Acids | 1.03 (0.98, 1.08) | 0.2521 | 1.03 (0.97, 1.09) | 0.3766 |
| Sodium | 0.96 (0.91, 1.01) | 0.119 | 0.97 (0.91, 1.04) | 0.4237 |
| Added sugar | 0.90 (0.84, 0.96) | 0.0007 | 0.89 (0.82, 0.96) | 0.0028 |
| Saturated Fatty Acids | 0.96 (0.91, 1.02) | 0.1859 | 0.95 (0.89, 1.02) | 0.1769 |
| **AHEI (Log2)** |  |  |  |  |
| Vegetable | 0.93 (0.89, 0.97) | 0.0009 | 0.95 (0.90, 1.00) | 0.0734 |
| Fruit | 0.94 (0.91, 0.97) | 0.0005 | 0.94 (0.89, 0.98) | 0.0034 |
| Whole grains | 0.95 (0.90, 1.00) | 0.0367 | 0.97 (0.91, 1.04) | 0.4221 |
| Nuts | 1.00 (0.95, 1.05) | 0.8928 | 1.04 (0.97, 1.11) | 0.2754 |
| Omega 3 fatty acid | 0.99 (0.95, 1.02) | 0.401 | 0.98 (0.94, 1.02) | 0.3773 |
| PUFA | 1.11 (1.00, 1.23) | 0.0403 | 1.12 (0.99, 1.27) | 0.077 |
| Total Meat | 0.97 (0.92, 1.02) | 0.2413 | 0.97 (0.91, 1.04) | 0.449 |
| Sodium | 1.01 (0.95, 1.08) | 0.7096 | 1.03 (0.96, 1.12) | 0.4187 |
| Alcohol | 0.91 (0.85, 0.98) | 0.0109 | 0.95 (0.86, 1.04) | 0.2812 |
| **DASHI (Log2)** |  |  |  |  |
| Fat | 0.94 (0.89, 0.98) | 0.004 | 0.94 (0.88, 0.99) | 0.0199 |
| Saturated fat | 0.96 (0.91, 1.02) | 0.1849 | 0.95 (0.89, 1.02) | 0.1868 |
| Protein | 0.94 (0.89, 0.98) | 0.0095 | 0.95 (0.89, 1.02) | 0.1773 |
| Cholesterol | 1.04 (0.98, 1.11) | 0.2036 | 1.05 (0.97, 1.13) | 0.2493 |
| Fiber | 0.94 (0.90, 0.98) | 0.0075 | 0.99 (0.93, 1.05) | 0.7463 |
| Magnesium | 0.91 (0.86, 0.96) | 0.0003 | 0.93 (0.86, 0.99) | 0.0341 |
| Calcium | 0.96 (0.92, 1.01) | 0.1422 | 0.97 (0.91, 1.04) | 0.3761 |
| Sodium | 0.94 (0.89, 0.99) | 0.0228 | 0.92 (0.86, 0.99) | 0.0176 |
| **MEDI (Log2)** |  |  |  |  |
| Olive oil | NA |  | NA |  |
| Whole fruits | 0.82 (0.41, 1.62) | 0.5691 | 0.84 (0.33, 2.09) | 0.7008 |
| Vegetables | 0.94 (0.67, 1.32) | 0.7265 | 0.74 (0.47, 1.15) | 0.1816 |
| Legumes | 0.88 (0.68, 1.14) | 0.3271 | 0.86 (0.61, 1.21) | 0.3896 |
| Nuts | 1.05 (0.85, 1.30) | 0.6303 | 1.22 (0.94, 1.58) | 0.1296 |
| Seafood | 0.68 (0.43, 1.08) | 0.0997 | 0.93 (0.55, 1.58) | 0.7996 |
| Alcohol | 0.85 (0.64, 1.13) | 0.2572 | 0.86 (0.60, 1.24) | 0.4152 |
| Sweets | 0.91 (0.76, 1.10) | 0.3361 | 0.98 (0.78, 1.24) | 0.8736 |
| Meats | 0.93 (0.80, 1.08) | 0.329 | 1.02 (0.84, 1.23) | 0.8539 |

Model 1 was adjusted for age, sex, race, marital status, education level, and poverty income ratio. Model 2 was adjusted for age, sex, race, marital status, education level, poverty income ratio, obesity, smoking, alcohol use, diabetes, high blood pressure, food insecurity, water consumption, serum calcium, and sitting time.

*HEI2020:

Total fruit: The serving size of total fruits including fruit juice, unit= cup eq.; Fruit: The serving size of Citrus, Melons, Berries + Other Intact Fruits, unit= cup eq.; Vegetable: The serving size of vegetables Total Vegetables + Legumes (Beans and Peas) in cup equivalents, unit= cup eq.; Green bean: The serving size of Dark Green Vegetables + Legumes (Beans and Peas) in cup equivalents, unit= cup eq.; Total Meat: The serving size of Total Meat, Poultry, and Seafood (including organ meats and cured meats) + Eggs + Nuts and Seeds + Soy + Legumes (Beans and Peas) in oz equivalents, unit=oz. eq., 1 cup legume = 4 oz; Seafood: The serving size of Seafood (high in n-3) + Seafood (low in n-3) + Soy + Nuts and Seeds + Legumes (Beans and Peas) in oz equivalents, unit=oz. eq., 1 cup legume = 4 oz; Whole grains: The serving size of whole grains, unit=oz. eq.; Dairy: The serving size of all dairy, unit=cup eq. Fatty Acids: The serving size of (Total Monounsaturated Fatty Acids + Total Polyunsaturated Fatty Acids)/Total Saturated Fatty Acids, unit=g; Sodium: The serving size of sodium, unit=mg; Added sugar: The serving size of added sugar, unit=tsp

AHEI:

Vegetable: The serving size of All vegetable except potatoes and legume, unit=servings/day (0.5 c of vege; 1 cup of green leafy (1 cup = 236.59 g); Fruit: The serving size of All whole fruits and no fruit juice, unit=servings/day (0.5 c of berries; 1 cup=236.59 g; 1 med fruit (1 cup = 236.59 g); Whole grains: The serving size of whole grains, unit=grams/day; Nuts: The serving size of Nuts, legumes, and vegetable protein (e.g., tofu), unit=servings/day = 1 srv=1oz (28.35 g) of nuts and legume or 1 TBLSP peanut butter (15 mL), 1 cup legume = 4 oz; Omega 3 fatty acid: The serving size of omega 3 fatty acid, unit=mg/day ( oz. = 28.35 g); PUFA: The serving size of PUFA, unit=% of energy; Polyunsaturated fatty acids (PUFAs); Total Meat: The serving size of red and processed meats, including Beef, pork, lamb, goat, veal, sausages, bacon, salami, ham, hot dog, deli meat, unit=servings/day; 1 srv= 4 oz. unprocessed meat; 1.5 oz. processed meat (1 oz. = 28.35 g); Sodium: The serving size of sodium, unit=mg/day per 2000 kcal; Alcohol: The serving size of alcohol, including Wine, beer, "light" beer, liquor, unit=drink/day (12 oz beer; 5 oz wine; 1.5 oz spirits) 1 oz = 28.35 g

DASHI:

Fat: The total fat intake, unit = g/day; Saturated fat: The saturated fat intake, unit = g/day; Protein: The protein intake, unit = g/day; Cholesterol: The cholesterol intake, unit = mg/day; Fiber: The fiber intake, unit = g/day; Magnesium: The magnesium intake, unit = mg/day; Calcium: The calcium intake, unit = mg/day; Sodium: The sodium intake, unit = mg/day.

MEDI:

Olive oil: The serving size of olive oil, unit= 10 g or 0.8 tbsp = 1 serving; Whole fruits: The serving size of All whole fruits, unit= 125 g = 1 serving (approximately 1 c); Vegetables: The serving size of All vegetables except potatoes and legumes, unit= 125 g = 1 serving (approximately 1 c of non-green leafy vege; 2 cup of green leafy vege); Legumes: The serving size of legumes, including Dried beans, lentils, peas, soups (split pea), tofu, soymilk, unit= 40 g = 1 serving (about 1.5 oz); Nuts: The serving size of nuts and seeds, including Peanuts, almonds, sunflower seeds, cashews, walnuts, unit= 25 g = 1 serving (about 1 oz); Seafood: The serving size of all fishes and seafoods, including Fresh-water and sea-water fish; preserved fish such as salted fish, canned fish; shellfish (squid, prawns, mollusks), unit=125 g = 1 serving (about 4 oz); Alcohol: The serving size of alcohol, including Wine, beer, "light" beer, liquor, unit= 14 g pure alcohol = 1 serving (12 ounces of beer, 5 ounces of wine, or 1.5 ounces of hard liquor, such as vodka or whiskey); Sweets: The serving size of all sweets, including Candy, chocolate, ice cream, cookies, cakes, pies, pastries, unit= 50 g = 1 serving (about 1.5 oz ) = 1 piece of candy, 1 chocolate bar, 1 scoop ice cream, 1 cookie, 1 cake slice, 1 pie slice, 1 pastry; Meats: The serving size of red and processed meats, including Beef, pork, lamb, goat, veal, sausages, bacon, salami, ham, hot dog, deli meat, unit= 150 g = 1 serving (about 5 oz)


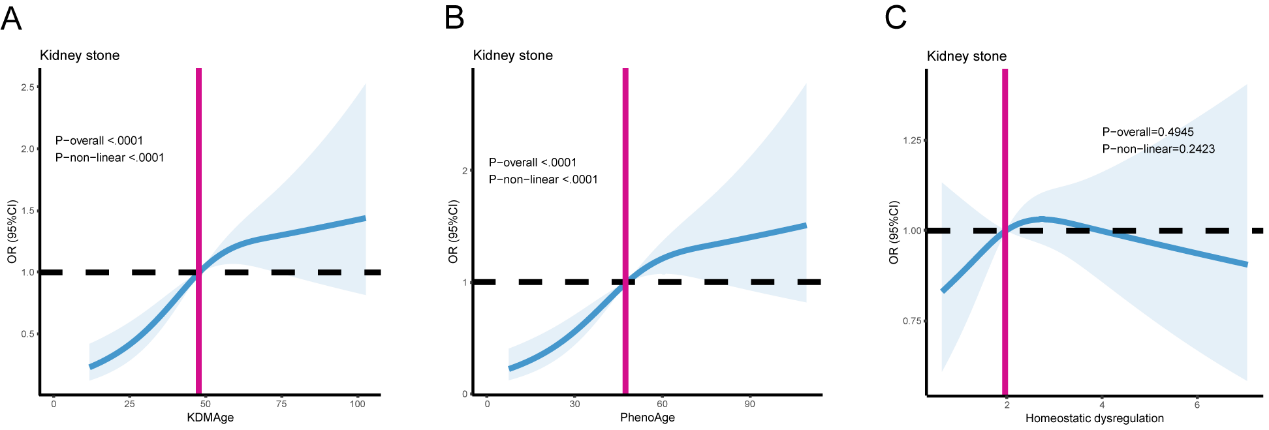


Figure S1 The non-linear associations between aging indicators and kidney stone by restricted cubic splines.

(A) The association between KDMAge and kidney stone; (B) The association between PhenoAge and kidney stone; (C) The association between homeostatic dysregulation and kidney stone.
